# Region-Specific Activation of *oskar* mRNA Translation by Inhibition of Bruno-Mediated Repression

**DOI:** 10.1371/journal.pgen.1004992

**Published:** 2015-02-27

**Authors:** Goheun Kim, Chin-I Pai, Keiji Sato, Maria D. Person, Akira Nakamura, Paul M. Macdonald

**Affiliations:** 1 Department of Molecular Biosciences, Institute for Cellular and Molecular Biology, The University of Texas at Austin, Austin, Texas, United States of America; 2 Laboratory for Germline Development, RIKEN Center for Developmental Biology, Kobe, Hyogo, Japan; 3 Proteomics Facility, Institute for Cellular and Molecular Biology and College of Pharmacy, The University of Texas at Austin, Austin, Texas, United States of America; 4 Department of Germline Development, Division of Organogenesis, Institute of Molecular Embryology and Genetics, Kumamoto University, Kumamoto, Japan; Harvard Medical School, Howard Hughes Medical Institute, UNITED STATES

## Abstract

A complex program of translational repression, mRNA localization, and translational activation ensures that Oskar (Osk) protein accumulates only at the posterior pole of the *Drosophila* oocyte. Inappropriate expression of Osk disrupts embryonic axial patterning, and is lethal. A key factor in translational repression is Bruno (Bru), which binds to regulatory elements in the *osk* mRNA 3′ UTR. After posterior localization of *osk* mRNA, repression by Bru must be alleviated. Here we describe an *in vivo* assay system to monitor the spatial pattern of Bru-dependent repression, separate from the full complexity of *osk* regulation. This assay reveals a form of translational activation—region-specific activation—which acts regionally in the oocyte, is not mechanistically coupled to mRNA localization, and functions by inhibiting repression by Bru. We also show that Bru dimerizes and identify mutations that disrupt this interaction to test its role *in vivo*. Loss of dimerization does not disrupt repression, as might have been expected from an existing model for the mechanism of repression. However, loss of dimerization does impair regional activation of translation, suggesting that dimerization may constrain, not promote, repression. Our work provides new insight into the question of how localized mRNAs become translationally active, showing that repression of *osk* mRNA is locally inactivated by a mechanism acting independent of mRNA localization.

## Author Summary

Proteins are often enriched to specific regions within cells via localization of mRNAs. This phenomenon serves a variety of roles, both bringing together factors involved in particular cellular processes to enhance their efficiency, and in restricting proteins that could do harm if deployed at inappropriate positions. In the latter situation, translational repression prevents expression before mRNA localization, and there must be activation mechanisms to inhibit or override repression. How the processes of mRNA localization and translation are coordinated is not well understood, in part because cellular extracts prepared to study mechanisms *in vitro* do not retain the spatial information present in the intact cell. We developed an *in vivo* assay to monitor the pattern of translation in the *Drosophila* oocyte, where several patterning determinants must be localized to specific regions. Using this assay, we showed that repression of translation by the Bruno protein is inhibited, and we could visualize when and where this occurs during oogenesis. Regional activation occurs not only at the site of mRNA localization, but more broadly in a graded fashion, and it does not require an activation element in the mRNA. We also show that Bruno dimerizes, and that dimerization is important for translational activation.

## Introduction

Localized mRNAs function in many biological settings to facilitate region-specific protein synthesis [1–3]. Translational repression of these mRNAs helps restrict distribution of the encoded proteins, an essential property if the protein has adverse effects at inappropriate locations. In addition, translational repression could be a prerequisite for mRNA localization, if the act of translation interferes with that process. With repression comes the need for translational activation, either by disrupting repression or by a separate mechanism. The *Drosophila oskar* (*osk*) mRNA is subject to an extensive program of regulation and provides a model for elucidation of the mechanisms of repression, localization and activation, and how these events are coordinated [4].

The Osk protein, whose distribution during oogenesis is restricted to the extreme posterior region of later stage oocytes, acts as a posterior determinant responsible for posterior patterning of the embryos and formation of the embryonic germline (reviewed in [5]). In the absence of Osk, the abdominal segments are missing and no germ cells form [6]. Conversely, mis- or overexpression of Osk, such that the protein is not tightly restricted to the posterior of the oocyte, leads to a reorganization of the embryonic body plan and ectopic formation of germ cells [7,8]. Thus, proper deployment of Osk is critical.

The *osk* mRNA is present from the earliest stages of oogenesis, transcribed in nurse cells and rapidly transported into the oocyte. As oogenesis proceeds, *osk* mRNA persists in the oocyte, and is transiently enriched near the anterior at stage 8 before assuming its final position at the extreme posterior of the oocyte [9,10]. It is only at this point that substantial levels of Osk protein accumulate [11–13]. The absence of Osk protein at earlier stages, and from unlocalized *osk* mRNA, is due to translational repression. A key player in repression is Bruno (Bru; encoded by the *aret* gene), which binds to multiple sites in the *osk* 3′ UTR. Mutation of these sites leads to excess Osk activity and precocious Osk protein [11]. Translational repression by Bru has been recapitulated using *in vitro* assay systems from *Drosophila* tissues [14,15].

Two models have been proposed for the mechanism of repression by Bru. In one model, the events that occur at the mRNA 5′ cap, a structure bound by eIF4E, are targeted. During cap-dependent initiation, eIF4E binds to eIF4G, resulting eventually in assembly of a functioning ribosome. The Cup protein also binds to eIF4E, using the same site bound by eIF4G. Another interaction of Cup is with Bru, leading to the model: Cup is recruited to an *osk* mRNA by Bru, binds to the eIF4E bound to the 5′ cap of that mRNA, and thus blocks the required eIF4E/eIF4G interaction [16]. In the other model for Bru-dependent repression, Bru promotes *osk* mRNA oligomerization and formation of large silencing particles, which are proposed to be inaccessible to ribosomes [17]. In this model Cup is also involved, but not the ability of Cup to bind eIF4E. Oligomerization of *osk* mRNA is also promoted by direct RNA dimerization and formation of large RNP complexes by Polypyrimidine Tract Binding protein (PTB) [18,19].

After the *osk* mRNA has been localized to the posterior pole of the oocyte at stage 9, translational repression must be overcome [11–13]. This could be achieved in different ways, with or without the need for specific regulatory elements. At one extreme, and requiring no activation elements, is inactivation or degradation of the repressors. At the other extreme, the repressors would remain in place and functional, but independent forms of activation (mediated by further regulatory elements) would overcome repression by exerting more powerful positive influences on translation. Just as for repression, there appear to be multiple contributions to activation: a variety of proteins and regulatory sequences have been implicated, with no unifying model for how their actions collectively lead to activation [11–13,20–26].

Here we characterize the interactions of Bru, using *in vitro* assays to map protein-binding domains and sites of phosphorylation, and designing mutations that affect the interactions. A simplified *in vivo* system, focusing on Bru-mediated regulation in the absence of much of the complex regulation of *osk* mRNA, reveals one form of translational activation and provides concrete insights into its mechanism.

## Results

### Bru dimerizes via a domain that also mediates Cup binding

**Fig 1:**
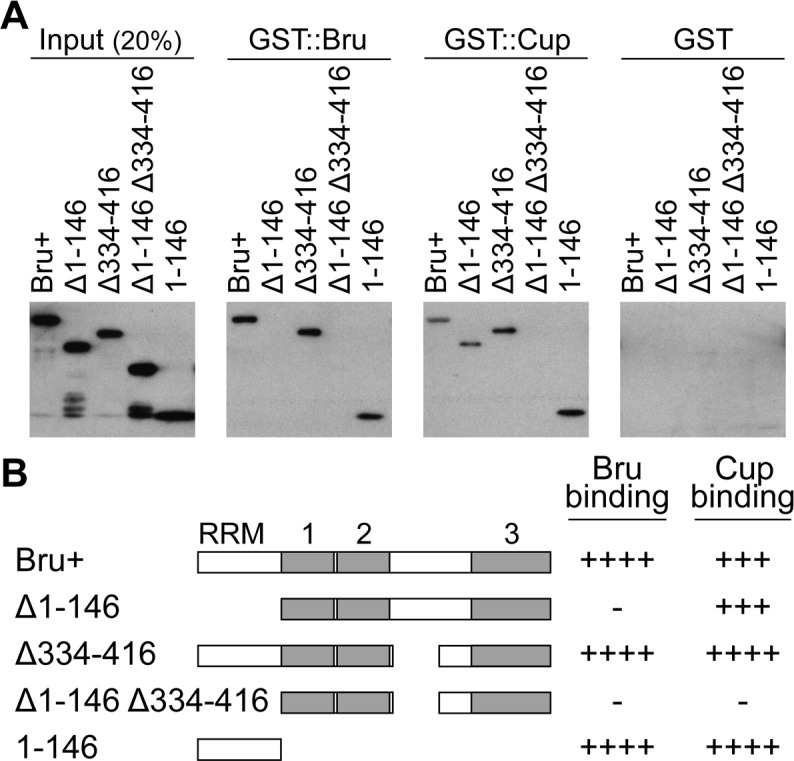
Bru domain 1–146 is important for both Bru and Cup binding. A. GST pull-down assays using the GST fusion proteins indicated at top, and the purified Bru proteins as labeled. Each panel is a Western blot probed with anti-His_6_ antibody, which detects the Bru proteins (but not GST::Bru). B. A schematic diagram of Bru proteins used in part A. The three RNA Recognition Motifs (RRMs) of Bru are shown as gray boxes; RRM3 is an extended RRM and is thus larger [59]. A summary of results from the pull-down experiments is shown on the right. ++++ indicates a wild-type level of binding,—is no detectable binding, and the intermediate values indicate the relative strengths of impaired binding.

A GST pull-down assay was used to test for the ability of Bru to dimerize. Full-length Bru was expressed as a fusion to GST, and incubated with Bru bearing a His_6_ tag. Following affinity purification of GST::Bru with glutathione sepharose beads, copurification of His_6_::Bru was tested by Western blot analysis using the anti-His_6_ antibody. By this assay, Bru did dimerize while His_6_::Bru did not bind to GST alone (Fig. 1A).

To map the domain of Bru responsible for dimerization, deletion derivatives of Bru (Fig. 1B) were tested in the GST::Bru pull-down assay. The three RRMs all function in RNA binding [27], so we focused on the other domains. Deletion of the Bru amino-terminal domain (aa1–146, Δ1–146) eliminated binding to GST::Bru, while deletion of most of the linker domain between RRMs 2 and 3 (aa334–416, Δ334–416) had no effect. The amino-terminal domain was not only required for dimerization with Bru, but was also sufficient: the isolated domain bound GST::Bru (Fig. 1A). The ability of Bru to dimerize provides an explanation for how Bru oligomerizes *osk* mRNA: a molecule of Bru bound to one *osk* mRNA could dimerize with a second molecule of Bru bound to a different *osk* mRNA. With the many Bru binding sites in the *osk* mRNA 3′ UTR [11,26], formation of large, highly interconnected protein-RNA assemblies is possible. This suggests that the proposed use of *osk* mRNA oligomerization as a mechanism of translational repression [17] would rely on Bru dimerization.

A second Bru interaction, with Cup, provides the basis for the other proposed mechanism of translational repression, in which Bru recruits Cup to the *osk* mRNA [16]. A GST::Cup pull-down assay was used to monitor interaction with Bru. As expected, full-length Bru (Bru+) bound GST::Cup. Deletion of either aa1–146 or aa334–416 of Bru had no dramatic effect on binding, but deletion of both domains eliminated binding. Just as for Bru dimerization, the isolated amino-terminal domain was sufficient for binding to GST::Cup (Fig. 1A,B).

### The amino-terminal domain of Bru is required for translational repression

Our evidence that the amino-terminal domain of Bru is essential for dimerization and contributes to Cup binding suggested that this domain is likely to play an important role in repression. To test this prediction we established an *in vivo* tethering assay, in which translation of a *GFP-MS2* reporter mRNA was monitored. The 3′ UTR of the *GFP-MS2* mRNA includes multiple copies of the bacteriophage *MS2* stem loop, a binding site for the MS2 coat protein (MCP [28]). Forms of Bru were expressed as fusions to MCP to direct binding to the reporter mRNA. Both the reporter mRNA and tethered Bru proteins were expressed in *Drosophila* ovaries using the UAS/GAL4 system.

**Fig 2:**
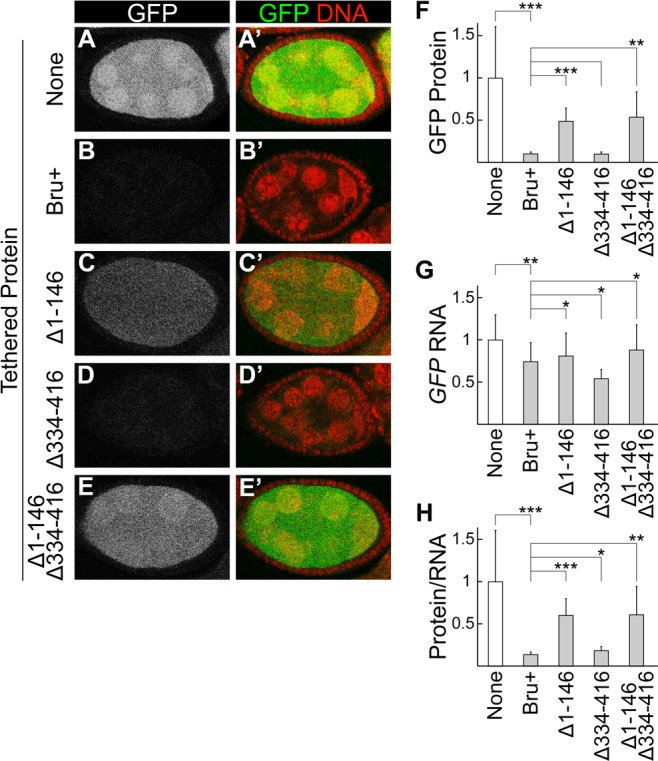
The Bru amino-terminal domain is essential for translational repression in a tethering assay. A-E, A’-E’. Egg chambers expressing the *GFP-MS2* reporter mRNA. (B-E, B’-E’) also express MCP::HA_3_::Bru proteins, of the type shown at left. All Bru proteins used include point mutations in RRM2 and RRM3 to inhibit RNA-binding activity, and thus prevent the early arrest of oogenesis caused by ectopic expression of Bru [27,60]. The RRM2 and RRM3 mutations have no effect on tethering, which relies on RNA binding by MCP. All samples were fixed in parallel and imaged together under the same settings. Expression of the UAS transgenes was driven by the *nosGAL4VP16* driver. Expression levels for different MCP::Bru proteins are shown in S1A Fig. F. GFP fluorescence was quantitated using Macnification and the value for none, which lacks any MCP::HA_3_::Bru proteins, was set to one. The mean and standard deviation were calculated from at least 40 samples per genotype. The asterisks indicate the Bru proteins with the GFP protein level differing significantly from the Bru+, using the student’s T test (**p≤0.01, ***p≤0.001). G. RNase protection assays: *GFP-MS2* RNA levels were quantified by ImageJ and normalized using the *rp49* signal. The value for none, which lacks any MCP::HA_3_::Bru proteins, was set to one. The mean and standard deviation were calculated from three independent experiments. The asterisks indicate the Bru proteins with the tethered *GFP* RNA level differing significantly from the Bru+, using the student’s T test (*p≤0.05, **p≤0.01). H. GFP fluorescence was normalized for the *GFP-MS2* RNA levels, which were normalized using the *rp49* RNA levels as in panel (G). The value for none, which lacks any MCP::HA_3_::Bru proteins, was set to one. The mean and standard deviation were calculated from at least 40 samples per genotype. The asterisks indicate the Bru proteins with the GFP protein/RNA level differing significantly from the Bru+, using the student’s T test (*p≤0.05, **p≤0.01, ***p≤0.001).

The *GFP-MS2* reporter by itself was expressed throughout the germline cells of the egg chamber (Fig. 2A). Coexpression of tethered Bru dramatically reduced the GFP level (10 fold; Fig. 2B,F). The strong reduction in GFP expression from tethered Bru was accompanied by a reduction in *GFP-MS2* mRNA level (1.7 fold; Fig. 2G). Therefore, in this assay Bru both repressed translation and reduced mRNA stability, although repression was the stronger effect (compare Fig. 2F and 2G).

Testing Bru mutants in the tethering assay revealed that deletion of the amino-terminal domain led to a substantial increase in GFP, although not to the level in the absence of repression (Fig. 2C,F). Deletion of the linker domain had no strong effect (Fig. 2D,F). The combination of deleting both the amino-terminal domain and the linker domain was no stronger than deleting the amino-terminal domain alone (Fig. 2F,H), but the range of fluorescence intensities was greater (error bars in Fig. 2F) and some egg chambers had weaker repression (Fig. 2E). Both mutants with enhanced GFP also had slightly elevated *GFP-MS2* mRNA (Fig. 2G), although the changes were strongest at the protein level (Fig. 2F,H). The differing activities of the mutant proteins were not due to differences in their expression (S1A Fig.). Thus, the mutants impaired both activities of Bru monitored in this assay: translational repression and destabilization of mRNA.

### Bruno is a phosphoprotein

**Fig 3:**
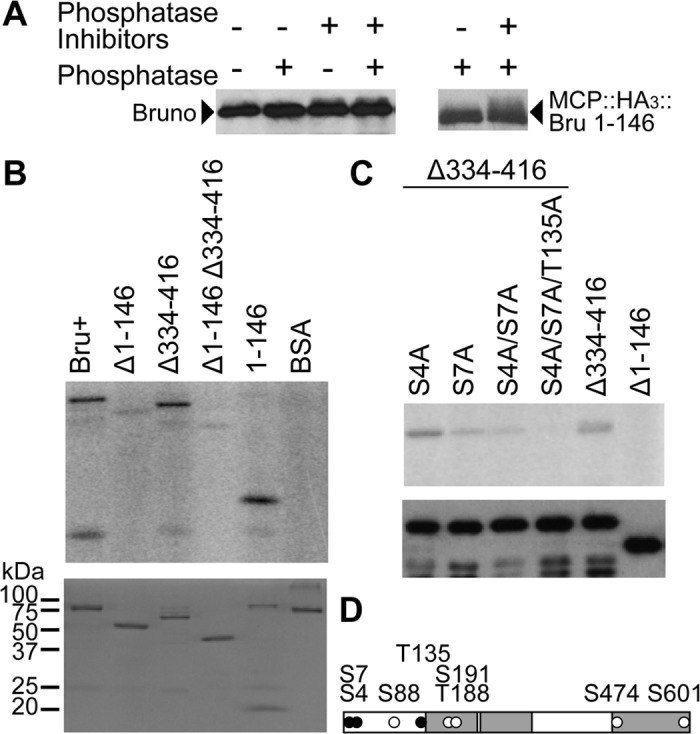
PKA phosphorylates Bru in the amino-terminal domain and Bru phosphosilent mutations disrupt phosphorylation by PKA. A. Left: Western blot of *wild-type* ovary extract after incubation with phosphatase and/or phosphatase inhibitors as indicated above. Proteins were detected using anti-Bru antibody. Right: Western blot of ovary extract from flies expressing MCP::HA_3_::Bru1–146 protein, with treatments noted as above. Proteins were detected using anti-HA antibody. Inhibitors used were sodium vanadate and beta-glycero phosphate, which are competitive inhibitors of the alkaline phosphatase. B. *In vitro* phosphorylation assay using gamma _32_P-ATP, purified mouse PKA catalytic subunit and purified Bru proteins as labeled (as in Fig. 1B). BSA was used as a negative control. Top: autoradiogram to detect phosphorylation. Bottom: Coomassie staining of proteins used for the phosphorylation assay to show the relative amounts of input proteins. The upper band in the 1–146 lane is a contaminating bacterial protein. C. *In vitro* phosphorylation assay using gamma _32_P-ATP, purified mouse PKA catalytic subunit and purified phosphosilent (Ala) mutant Bru proteins as labeled. The positions of amino acids predicted to be candidates for phosphorylation by PKA are shown in the schematic (D). The point-mutated Bru proteins have the Δ334–416 deletion, which does not affect phosphorylation (panel B). Top: autoradiogram to detect phosphorylation. A similar assay using the same mutations in the context of the full-length Bru is shown in S4B Fig. Bottom: Western blot of proteins used in the phosphorylation assay to show the relative amounts of input proteins. D. A schematic diagram of Bru showing PKA phosphorylation sites predicted by NetPhosK and KinasePhos. Three amino acids, S4, S7 and T135, depicted as black circles, were tested in different experiments by mutating them to either alanine (phosphosilent) or glutamate (phosphomimetic).

To allow translation of the *osk* mRNA once it has been localized to the posterior pole of the oocyte, there must be a release from repression. How this is accomplished is not known, but one possibility is that Bru is post-translationally modified to change its activity. To ask if Bru is phosphorylated, the conventional approach of testing for phosphatase-dependent changes in electrophoretic mobility of the protein was used. In untreated ovary extract, Bru appeared by Western blot analysis as a major band, with a faint lower-mobility band. Treatment with phosphatase eliminated the weak band. By contrast, addition of phosphatase inhibitors enhanced the minor band, consistent with the interpretation that this small fraction of Bru is phosphorylated (Fig. 3A left).

To make a more compelling case for phosphorylation, we also tested the MCP::HA_3_::Bru 1–146 protein from above, which at 32 kDa is substantially smaller than Bru (64 kDa) and thus might display a larger change in mobility from phosphorylation. This was indeed the case, and the difference between the major Bru band and the slower migrating fraction was more dramatic (Fig. 3A right). Bru phosphorylation was also analyzed by phosphate-affinity SDS-PAGE with the acrylamide-pendant Phos-tag, which separates different phosphoprotein isoforms [29]. Using this approach, multiple, different phosphorylated species could be detected (S2A Fig.).

### Bruno is phosphorylated by PKA

The NetPhosK 1.0 and KinasePhos prediction programs were used to identify candidate phosphorylation sites in Bru. Both report multiple sites for many different kinases, although none of the candidate sites had scores suggesting a high probability of phosphorylation (S1 Table). Nevertheless Bru is phosphorylated, and so even the sites with modest scores remain as candidates. Several amino acids are predicted to be targets for Protein Kinase A (PKA), an interesting option since alteration of PKA activity affects *osk* expression pattern and embryonic body patterning [30].

To evaluate PKA, *in vitro* phosphorylation assays were performed using the PKA catalytic subunit and full-length Bru. Bru was strongly phosphorylated, while BSA (a negative control) was not (S3A Fig.). By contrast, neither Casein Kinase I (CK1) nor Calmodulin-dependent Protein Kinase II (CaMKII), which share a part of the recognition motif of PKA [31–33], supported detectable phosphorylation of full-length Bru (S3B Fig.).

To map the sites of phosphorylation, Bru deletion proteins from above (Fig. 1B) were used as substrates for PKA. Deletion of the amino-terminal domain greatly reduced phosphorylation, and the isolated domain was itself phosphorylated. Deletion of the linker region did not reduce phosphorylation, nor did it enhance the effect of deleting the amino-terminal domain (Fig. 3B). Thus, PKA phosphorylates the amino-terminal domain of Bru.

To identify sites of phosphorylation, we performed a tandem mass spectrometry (MS/MS) analysis of Bru phosphorylated *in vitro*. Although four candidate sites are predicted in the amino-terminal domain, only phosphoserine at position 7 (S7) was identified with high confidence and no ambiguity (Fig. 3D). S88 could not be tested since aa36–119 was undetectable due to a low coverage of MS/MS (see Materials and Methods), and a majority of peptides containing either S4 or T135 was detected as unphosphorylated. Nevertheless, there is still a possibility of weak phosphorylation below the limit of detection at either S4 or T135. Mutation of S7 to alanine (S7A) substantially reduced phosphorylation by PKA. Because the S7A mutant retained a low level of phosphorylation, we also tested mutations in the other predicted sites, either alone or in combinations. Of the mutants tested, S4A/S7A/T135A was most resistant to phosphorylation (Fig. 3C). The mutants, except for S4A, were also tested in the context of full-length Bru, and similar results were obtained (S2B Fig.).

### Phosphomimetic mutations prevent Bru dimerization and impair Cup binding

Since the amino-terminal domain of Bru is essential for repression, the potential phosphorylation of one or more residues within this region might inhibit or enhance repression. We therefore asked if phosphomimetic mutations would interfere with Bru protein interactions mediated by the amino-terminal region and implicated in repression.

**Fig 4:**
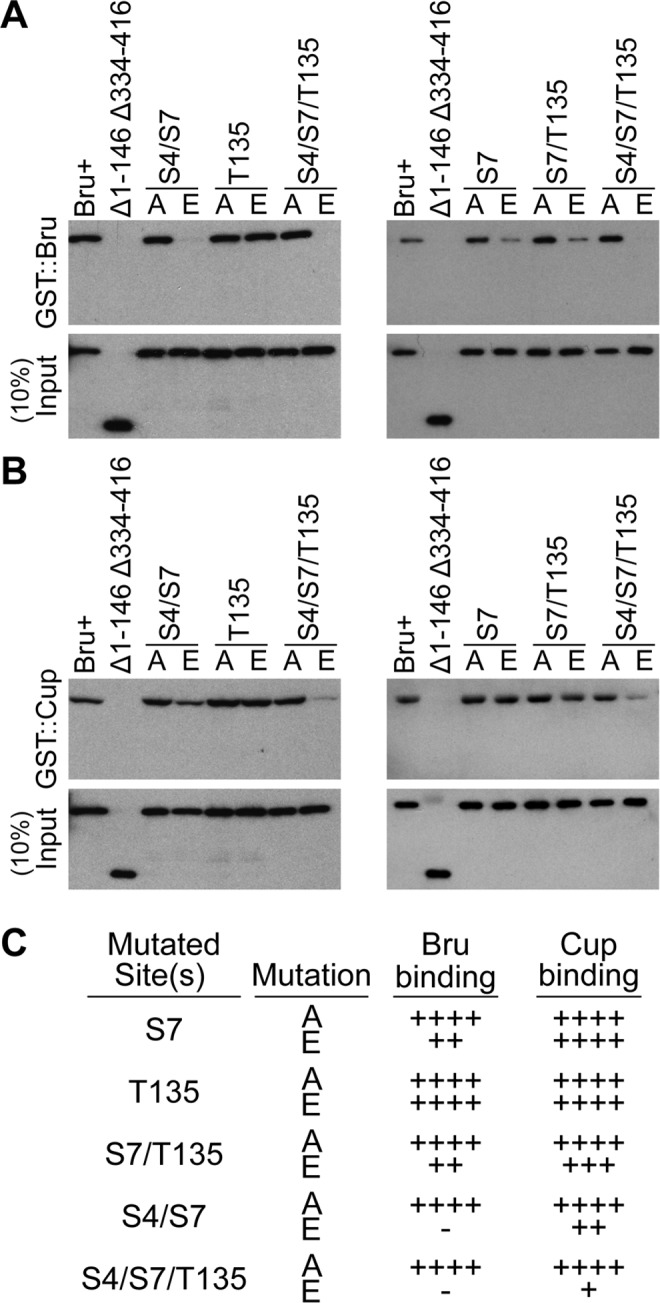
Bru phosphomimetic mutations additively impair both Bru-Cup and Bru-Bru interactions. GST::Bru (A) and GST::Cup (B) pull-down assays to detect interactions with Bru proteins. The input proteins are indicated at top, with Δ1–146 Δ334–416 as a negative control. Each panel is a Western blot probed with anti-His_6_ antibody, which detects the Bru proteins (but not GST::Bru). C. Summary of results from the pull-down assay. ++++ indicates a wild-type level of binding,—is no detectable binding, and the intermediate values indicate the relative strengths of impaired binding.

Pull-down assays were performed with GST::Bru and GST::Cup, using Bru mutants with phosphosilent alanine (A) or phosphomimetic glutamate (E) substitutions at one or more of the three residues that affect phosphorylation by PKA: S4, S7, and T135. None of the phosphosilent mutants showed reduced binding to GST::Bru (Fig. 4A) or GST::Cup (Fig. 4B), demonstrating that mutation of the affected residues did not inherently disrupt the protein interactions. By contrast, the phosphomimetic mutants altered interactions. S7E significantly reduced dimerization, and the S4E/S7E double mutant retained only a very low level of dimerization. Including the T135E mutation did not obviously further reduce dimerization by S7E (in S7E/T135E), but did reduce dimerization in the triple mutant (S4E/S7E/T135E) to below the level of detection (Fig. 4A and 4C).

Bru binding to Cup was less sensitive to the phosphomimetic mutations. S7E did not reduce binding, and the double mutation combinations caused only modest defects. Even the S4E/S7E/T135E triple mutant retained detectable binding (Fig. 4B and 4C).

Because the S4E/S7E/T135E triple mutant eliminated detectable Bru dimerization, repression dependent on this interaction is expected to be disrupted. The prediction is less clear for Cup-dependent repression, given the residual Bru/Cup interaction.

### Mutations that prevent Bru dimerization do not affect repression

**Fig 5:**
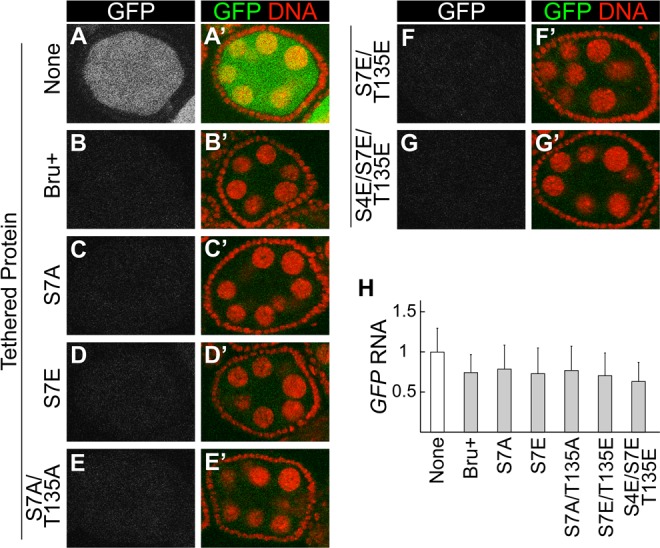
Interaction-defective Bru mutants retain strong repressive activity in the tethering assay. A-G, A’-G’. Egg chambers expressing the *GFP-MS2* reporter mRNA. (B-G, B’-G’) also express MCP::HA_3_::Bru proteins, of the type shown at left. All Bru proteins include point mutations in RRM2 and RRM3 (see Fig. 2 legend). All samples were fixed in parallel and imaged together under the same settings. Expression of the UAS transgenes was driven by the *nosGAL4VP16* driver. The MCP::HA_3_::Bru proteins were expressed at similar levels, except for S4E/S7E/T135E which was slightly elevated (S1B Fig.). H. RNase protection assays: *GFP-MS2* RNA levels were quantified by ImageJ and normalized using the *rp49* signal. The value for none, which lacks any MCP::HA_3_::Bru proteins, was set to one. The mean and standard deviation were calculated from three independent experiments.

Our analysis of PKA phosphorylation of Bru *in vitro* suggests that PKA may also modify the protein *in vivo*. Testing this prediction has proven to be challenging, with no conclusive answer. This is due in part to failure in making an antibody against the phospho-S7 peptide. Nevertheless, the Bru mutants defective in dimerization provide useful tools to test the importance of this interaction for Bru function. As one such test, we made use of the tethering assay to monitor translational repression. Notably, none of the mutants tested, including the S4E/S7E/T135E triple mutant that prevented Bru dimerization and impaired the Bru/Cup interaction, showed any substantial decrease in repression (Fig. 5). Likewise, there were no substantial changes in reporter mRNA levels (Fig. 5H).

MCP is known to dimerize [34]. This property could substitute for Bru dimerization, and thus neutralize the effect of the dimerization-defective mutations. To address this possibility, we tested another tethering system which relies on binding of a bacteriophage lambda N peptide (which does not dimerize) to the *boxB* stem-loop RNA [35]. In this case, the reporter mRNA was *GFP* with 6 copies of the *boxB* sequence in the 3′ UTR (*GFP-boxB*), and the Bru proteins were expressed as fusions to the λN peptide. Just as with the other system, tethered Bru repressed translation of the reporter mRNA (compare S4A and S4B Fig.). Notably, the S4E/S7E/T135E triple mutant did not affect repression (S4C,D Fig.), confirming that dimerization is not required for translational repression.

### Dimerization-defective Bru mutants have weakly impaired RNA-binding activity

Cooperative binding is a common strategy to enhance affinity for a substrate. Dimerization of Bru might facilitate cooperative binding to RNA, and if so, the mutations inhibiting dimerization are expected to impair RNA-binding activity of Bru. A UV-crosslinking assay was used to test Bru proteins for their ability to bind the *osk* 3′ UTR *AB* region RNA, which has multiple Bru binding sites [11]. The two mutants most strongly defective in dimerization, S4E/S7E and S4E/S7E/T135E, showed compromised RNA binding (S5A Fig.). After quantitation, normalization for protein levels and statistical analysis of three independent experiments, both mutants were considered to have a significant change in their RNA-binding ability when compared to their ala-mutant (dimerization-competent) counterparts (S5B Fig.). Although the reduction in RNA binding is modest, it is possible that this change could contribute to a reduction in Bru activity *in vivo* by weakening the interaction with target mRNAs, such as *osk*.

### A reporter mRNA to detect the pattern of translation *in vivo*

The tethering assays were useful for monitoring, in isolation from much of the complex program of *osk* regulation, translational repression by Bru. However, these assays have limitations. First, any region-specific change in repressive activity might be missed, as the reporter protein is diffusible. Second, since the RNA-binding activity of Bru was not required in the assay, a possible disruption of repression by reduced RNA-binding affinity would not be detected.

As an alternate approach to address these limitations we wanted to maintain the use of a simplified system which focuses on repression by Bru, but allows detection of regional differences in translation under conditions where Bru binds directly to the mRNA. Our approach has two components. The first, described in the following paragraphs, is the development of an appropriate reporter mRNA. The second, described in the next section, is manipulation of the *aret* gene, which encodes Bru, to introduce the mutations that disrupt Bru dimerization.

**Fig 6:**
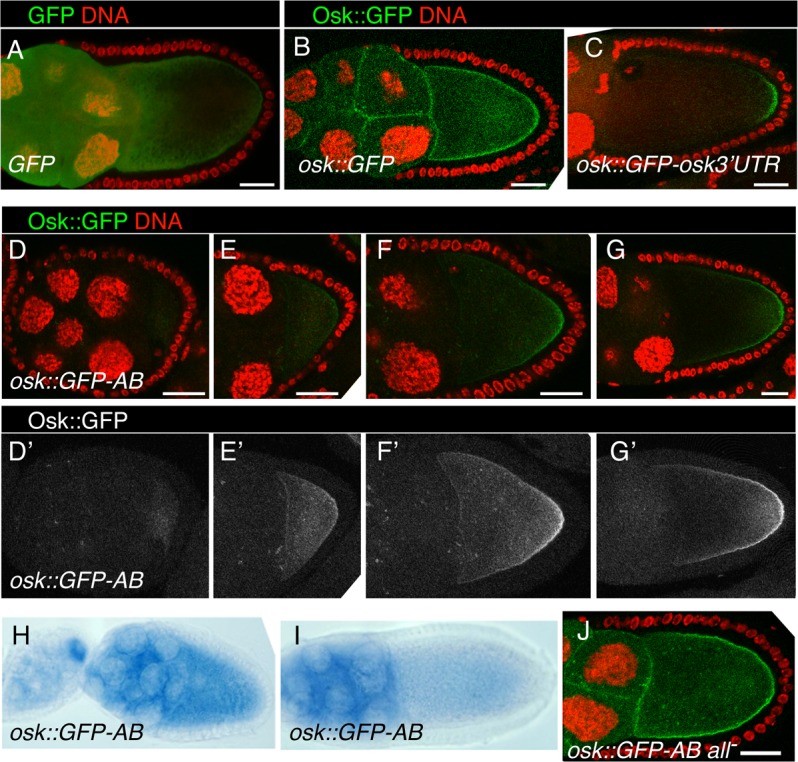
Regional inhibition of Bru repression. A-C. Distribution of GFP fluorescence from *UAS-GFP* (A), *UAS-osk1–534*::*GFP* (B) and *UAS-osk1–534*::*GFP-osk3′UTR* (C). For this and all panels except D′-G′ and H and I, GFP fluorescence is green and DNA (ToPro-3 staining) is red. For all panels, transgene expression was driven with the *matα4-GAL-VP16* driver and scale bars are 25 μm. D-G. Distribution of GFP fluorescence from *UAS-osk1–534*::*GFP-AB* at progressively later stages of oogenesis. In panels D′-G′, only the GFP channel is shown, with the signal enhanced (identically for all) in Adobe Photoshop to better reveal loss of repression in the oocyte. At the earliest stage shown (D,D′), there is a very low level of Osk1–534::GFP distributed throughout the oocyte. Later, the oocyte signal increases, initially ungraded (E,E′) and later clearly in a gradient from the posterior (F,F′,G,G′). H,I. Distribution of *osk1–534*::*GFP-AB* mRNA, detected by in situ hybridization. mRNA signal is blue. J. Loss of translational repression from *UAS-osk1–534*::*GFP-AB all-*. No posterior gradient of GFP can be detected.

A requirement for the reporter mRNA is that the encoded protein be anchored, such that its site of synthesis is revealed. The Osk protein is itself anchored to the oocyte cortex, with the amino-terminal domain of the Long Osk isoform required for this function [36]. To determine if this domain would anchor GFP, the *UAS-osk1–534*::*GFP* transgene (which includes nucleotides 1–534 of the *osk* mRNA, and thus the first 173 amino acids of Long Osk) was tested. While GFP alone appears diffuse throughout the germline cells (Fig. 6A), Osk::GFP is highly enriched at cortical regions of the oocyte and at cell boundaries in the nurse cells (Fig. 6B). Addition of the *osk* 3′ UTR to this transgene (in *UAS-osk1–534*::*GFP-osk3′UTR*) restricts expression to the posterior pole of the oocyte, where the mRNA is localized. Notably, the protein from *osk1–534*::*GFP-osk3′UTR* mRNA remains concentrated in a narrow crescent at the posterior pole, and does not diffuse extensively along the oocyte cortex (Fig. 6C). Therefore, the amino-terminal domain of Osk provides a useful anchoring domain to reveal the site of translation.

The reporter mRNA also needs to be subject to Bru-dependent repression, and with Bru binding directly to the mRNA. Just as in the tethering assay, we wanted to avoid the complexity of the full program of *osk* regulation. This can be achieved through the use of the *osk* 3′ UTR AB region, which contains multiple Bru binding sites [11,26]. Addition of this region to a GFP reporter confers efficient repression in the ovary [26], and as expected repression was dependent on the Bru binding sites (below).

Combining these two required features for the reporter mRNA, the *UAS-osk1–534*::*GFP-AB* transgene encodes an anchored GFP protein whose translation will be repressed by Bru.

### Region-specific activation of *osk* mRNA translation

Characterization of the *osk1–534*::*GFP-AB* reporter mRNA confirmed the expected translational repression, but also revealed a novel pattern of region-specific translational activation.

During previtellogenic stages of oogenesis the *osk1–534*::*GFP-AB* mRNA was strongly repressed throughout the egg chamber, with no detectable GFP signal (Fig. 6D). Translation of *osk1–534*::*GFP-AB* mRNA continued to be strongly repressed in nurse cells at later stages of oogenesis. However, starting at stage 7/8 a faint Osk::GFP signal was detected in the oocyte (Fig. 6E). The intensity of the signal increased at later stages and, strikingly, Osk::GFP accumulated in a weak gradient extending from the posterior pole (Fig. 6F,G). This mRNA lacks localization signals required for posterior localization of *osk* mRNA [37,38], and, not surprisingly, we found no detectable posterior localization of the reporter mRNA (Fig. 6H,I). Therefore, translation of the reporter was activated independent of association with the mRNA localization machinery.

There are two options to explain the posterior gradient of the anchored Osk::GFP fusion protein. One is that activation of translation occurred in a posterior gradient essentially the same as that displayed by Osk::GFP. Alternatively, activation of translation could have occurred just where pole plasm assembles at the posterior pole of the oocyte (from the fraction of the mRNA located in that region by chance), followed by diffusion of Osk:GFP from that site to create the extended gradient we observe. However, as shown above, when we selectively expressed the same Osk::GFP fusion protein just at the posterior pole from a localized mRNA, it remained there and did not form a gradient (Fig. 6C). Thus, the gradient of Osk::GFP produced by the unlocalized transgene mRNA must have formed by translational activation in a broad and graded domain: highest at the posterior pole, extending with diminishing strength along almost the entire length of the oocyte, and virtually undetectable at the anterior margin of the oocyte.

These results reveal a spatially-restricted form of translational activation, which we call region-specific activation. This form of activation could inhibit Bru-mediated repression. Alternatively, activation could be independent, and simply superimposed on repression. To distinguish between these options we tested a version of the transgene, *UAS-osk1–534*::*GFP-AB all-*, with the Bru binding sites mutated. The mutations disrupted repression, allowing translation of the Osk::GFP protein, which appeared in nurse cells and oocytes (Fig. 6J). If activation inhibits repression by Bru, in the absence of repression there would be no detectable activation and the Osk::GFP protein should be present at similar levels along the anteroposterior axis of the oocyte. By contrast, if activation is independent of Bru, it should still occur when the Bru binding sites are mutated, leading to a higher level of Osk::GFP in the more posterior portion of the oocyte. We detected no posterior enhancement of Osk::GFP when repression was disrupted (Fig. 6J). Thus, region-specific activation of translation must act by inhibiting the repressive function of Bru.

### Dimerization-defective Bru mutants have impaired region-specific activation of translation

**Fig 7:**
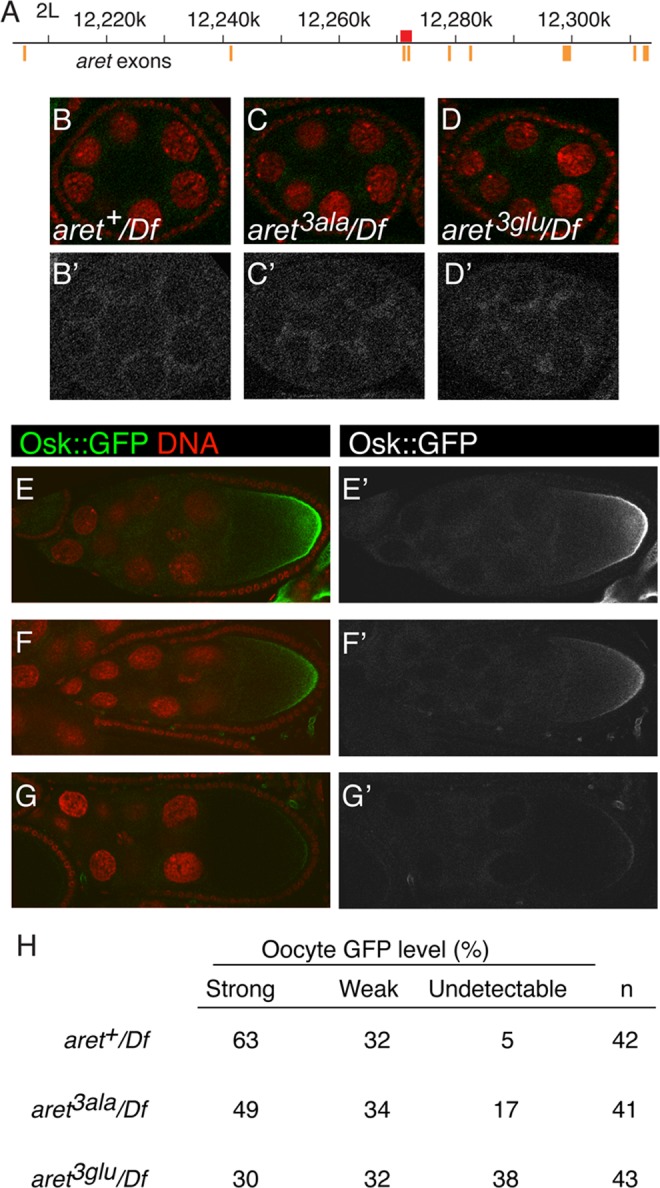
Translational repression and activation in *aret* mutants. A. Schematic diagram of the *aret* locus. Numbering is according to the *Drosophila* genome sequence, R5.48. Orange bars depict exons (the widths of the bars are not to scale), and the red rectangle is the 2.1kb targeted region, which when translated, includes the amino-terminal domain of female Bru.B-D, B’-D’. Stage 6 egg chambers expressing the *osk1–534*::*GFP-AB* reporter in different *aret* mutant backgrounds, as labeled. *Df* is *Df(2L)BSC407*. For B-D GFP is in green and nuclei in red. Panels B′-D′ show just the GFP channel. E-G, E’-G’. Examples of phenotypic categories for panel H. For E-G GFP is in green and nuclei in red. Panels E′-G′ show just the GFP channel. All samples were fixed in parallel and imaged together under the same settings. Expression of the UAS transgene was driven by the *matα4-GAL-VP16* driver. H. Intensity of the posterior zone of GFP from *UAS-osk1–534*::*GFP-AB* in *aret* mutants. Examples of strong, weak and undetectable are shown in E/E′, F/F′ and G/G′, respectively.

**Fig 8:**
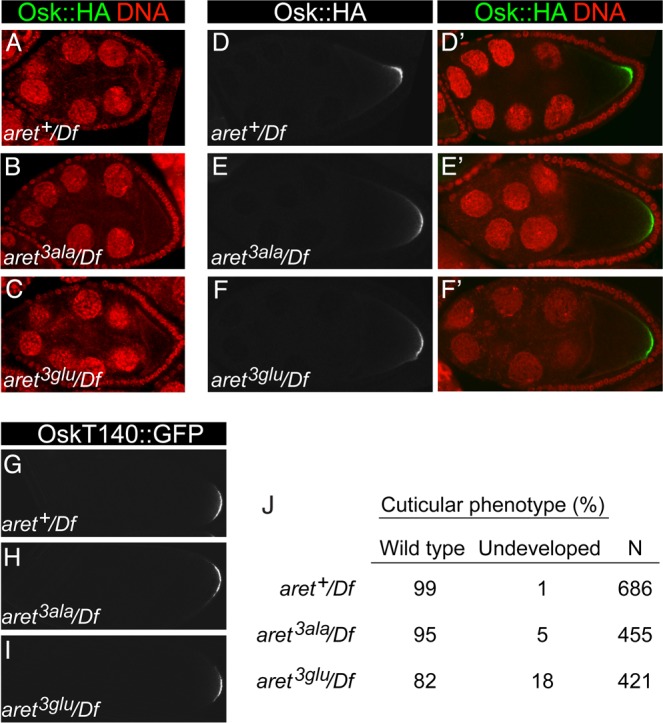
Osk expression in *aret* replacement lines. A-F, D’-F’. Egg chambers expressing the *oskT140*::*HA* genomic transgene (whose expression mimics that of *osk*, Methods and Materials), in different genetic backgrounds with engineered *aret* alleles, as labeled. All have a single copy of *aret* in *trans* to *Df(2L)aret* and two copies of *oskT140*::*HA*. (A-C) are stage 8, and (D-F) are stage 9 egg chambers. (D’-F’) show Osk::HA in green and nuclei in red. All samples were fixed in parallel and imaged together under the same settings. G-I. Late-stage egg chambers expressing the *oskT140*::*GFP* genomic transgene (whose expression mimics that of *osk*) in different genetic backgrounds with engineered *aret* alleles, as labeled. All have a single copy of *aret* in *trans* to *Df(2L)aret* and two copies of *oskT140*::*GFP*. All samples were fixed in parallel and imaged together under the same settings. J. Cuticular phenotypes of embryos from *aret* mutant mothers. All have *aret* in *trans* to *Df(2L)aret*. *aret^+^* is the *wild-type* replacement.

For the assay system showing region-specific activation of translation, Bru is provided by the endogenous gene. To test dimerization-defective Bru in this assay, we used homologous recombination (HR) [39] to exchange exons that encode the amino-terminal region of Bru (Fig. 7A). The replacements were *wild type* (*aret^+^*), *S4A/S7A/T135A* (*aret^3ala^*), or *S4E/S7E/T135E* (*aret^3glu^*). Loss of *aret* function leads to an early arrest of oogenesis, with no oocyte specified [40]. Females in which the HR replacement alleles provided the only copy of *aret* all displayed normal progression through oogenesis, indicating that the mutations did not substantially alter *aret* function. Protein levels for the different alleles were similar (S6 Fig.). Each of the proteins showed the normal distribution of Bru (S6 Fig.), including the granular cytoplasmic appearance due to association with nuage and sponge bodies [41]. Embryos obtained from these females were tested for patterning defects. Although misregulation of *osk* expression—either too little or too much Osk activity—causes striking patterning defects [6–8], no such defects were found for any of the *aret* HR alleles. The only phenotype detected was for the *aret^3glu^* mutant, and was unrelated to any known *osk* defect: an increase in the proportion of embryos that fail to develop (Fig. 8J).

To ask if preventing dimerization of Bru influenced the regional activation of translation, expression of the *osk1–534*::*GFP-AB* mRNA was monitored in the engineered *aret*-mutant ovaries (Fig. 7B-G). Repression in nurse cells was similar for all genotypes, consistent with the absence of any repression defect in the tethering assays (Fig. 7B-D). Both *aret^3ala^* and *aret^3glu^* mutants reduced the degree of posterior activation, with the strongest effect from the Bru dimerization- defective *aret^3glu^* mutant: the fraction of oocytes with a strong posterior gradient was about half that of *aret^+^*, and over a third of the oocytes had no detectable posterior gradient (Fig. 7H). Thus, the *aret^3glu^* mutant substantially disrupted regional activation. Because this mutant, in effect, enhanced repression, these results are consistent with the failure to detect any loss of repression in the tethering assays.

The *aret^3ala^* and *aret^3glu^* mutants were also tested for an effect on Osk expression. Because defects in activation of *osk* mRNA translation can be most pronounced late in oogenesis [26], this analysis included the use of a genomic *osk*::*GFP* transgene for detection of Osk::GFP after deposition of the vitelline membrane (which is impermeable to antibodies), as well an epitope tagged *osk* transgene (*oskHA*) for detection at earlier stages. There was no defect in repression in stage 8 egg chambers (Fig. 8A-C), as expected from the tethering assay results. Likewise, we did not detect any difference in Osk expression pattern among the three replacement lines in stage 9 (Fig. 8D-F) or later (Fig. 8G-I) egg chambers, consistent with the absence of patterning defects (Fig. 8J) and suggesting redundancy in mechanisms of translational activation.

## Discussion

To better understand translational regulation of *osk* mRNA by Bru, we characterized Bru protein interactions and developed *in vivo* assays to monitor Bru activity. Bru binds Cup, an interaction that is essential for at least one model of repression [16]. Here we have shown that Bru dimerizes, an interaction very likely to contribute to a second model of repression. In that model, the oligomerization of *osk* mRNA by Bru creates silencing particles, in which *osk* transcripts are made inaccessible to the translation machinery. The evidence for this model comes from an *in vitro* assay using purified Bru, which oligomerizes RNAs containing many Bru binding sites [17]. Because no other macromolecules are present in the assay, dimerization of Bru appears to be the only plausible option for oligomerization of the RNAs. The alternative is for the different Bru RNA-binding domains (there are three RRMs [27,42,43]) to bridge different RNAs, a kinetically unfavorable option: after the initial binding of one Bru domain to an RNA (a second order reaction), the subsequent binding of another Bru domain would strongly favor association with the same RNA (first order) as opposed to another RNA (second order). Furthermore, only one of the RRMs provides a high degree of binding specificity [43], and so specificity of oligomerization would be low (and inconsistent with the observed binding specificity of the intact protein [11,43]) if different domains bound to different RNAs.

We found that the amino-terminal domain of Bru is essential for dimerization. The same domain was sufficient for Cup binding, and deletion of both the amino-terminal and linker domains was required to eliminate Cup binding. These Bru/Cup interaction data using a GST pull-down assay contrast with those from the yeast two-hybrid assay, where the linker domain was shown to be both necessary and sufficient for Cup interaction, while the amino-terminal domain was neither necessary nor sufficient [16]. We do not know why there are differences. In both assays Bru is present as a protein fusion, and it may be that the different additional protein domains—GST in the one assay, and a transcriptional activation domain in the other assay—differentially constrain Bru binding.

To explore the role of dimerization on Bru activity *in vivo*, two types of simplified assays were used, both of which monitor the activity of Bru independent of much of the rest of the complex regulation of *osk* mRNA. In one type of assay, Bru was tethered to a reporter mRNA whose repression was monitored. For this assay, a variety of Bru mutants were used, both deletions and point mutations. In this assay the amino terminus of Bru was shown to be essential for repression, while the point mutants that prevent Bru dimerization and reduce Bru binding to Cup had no effect on repression. Therefore, Bru dimerization was not essential for repression. Notably, the tethering assay also revealed that Bru binding reduced stability of the reporter mRNA, in addition to translational repression. Regulation by Cup is known to alter mRNA stability in cultured cells [44], and our results are consistent with that property.

The second type of simplified *in vivo* assay made use of a reporter mRNA designed to display the pattern of translation within cells, not simply the total level of the encoded protein. Strikingly, Bru-dependent repression of the reporter mRNA was alleviated within the oocyte in a graded fashion, with peak translation at the posterior pole of the oocyte and extending with decreasing efficiency most of the distance to the anterior of the oocyte. Thus, this reporter revealed one form of translational activation—region-specific activation—for which concrete conclusions about mechanism can be drawn: activation is regional, but not narrowly restricted to the site at which *osk* mRNA is localized; activation is not mechanistically coupled to mRNA localization; and activation involves the inhibition of Bru repression.

To test the requirement for Bru dimerization in region-specific activation, homologous recombination was used to introduce point mutations into the *aret* locus. The new alleles can be used with no concerns about appropriateness of expression or artifacts from use of fusion proteins. Notably, the mutations preventing dimerization interfered with region-specific activation, suggesting that dimerization acts to disrupt repression, opposite of what might be expected for the silencing particles model of repression.

Region-specific activation disrupts or alters Bru activity, which could involve a direct effect on Bru, or be mediated by a regulatory element which could indirectly affect Bru. A direct effect on Bru could entail, for example, phosphorylation. We have shown that PKA phosphorylated Bru *in vitro*, and PKA plays a positive role in Osk expression [30]. PKA is involved in a posterior signal transduction event in the oocyte [45], and could thus act in the region where activation occurs (although there has been no demonstration of the site of PKA activity). However, phosphomimetic mutations of the sites at which PKA phosphorylates Bru *in vitro* did not promote activation, and instead diminished activation. Nevertheless, phosphorylation or another post-translational modification remains as a likely option for region-specific activation, as observed for other repressors of localized mRNAs in which this modification reduces RNA binding affinity [46–48]. The mutations mimicking phosphorylation of Bru also reduced RNA binding affinity, but by a modest degree and the significance of this effect is uncertain. It is noteworthy that region-specific activation was not tightly confined to the extreme posterior of the oocyte. This very strongly indicates that the factors involved are not restricted to the germ plasm that assembles in a crescent at the posterior pole.

If region-specific activation relies on a regulatory element, it must lie within the *osk* sequences of the *osk1–534*::*GFP-AB* mRNA, either the 5′ *osk* sequences or the 3′ UTR AB region. There has been no indication of a role for the AB region in translational activation. The 5′ *osk* sequences have been reported to include a translation activation element which mediates posterior-specific inhibition of Bru-mediated repression [21]. However, recent work shows that the 5′ element is not essential, and that the evidence for it functioning only at the posterior of the oocyte is based on an incorrect assumption (M Kanke and PMM, submitted).

Multiple factors and *cis*-acting elements have been implicated in translational activation of *osk* mRNA [11–13,20–26], and it is clear that the region-specific mechanism identified here is not the only form of activation. Indeed, the mutant *aret* alleles which disrupt regional activation of the *osk1–534*::*GFP* reporter mRNA have no detectable effect on regulation of endogenous *osk* mRNA. This is most likely due to redundancy, with the loss of one form of activation being obscured by persistence of other types of activation. Redundancy in activation mechanisms is not unexpected: there are multiple contributions to repression of *osk* mRNA translation [11,16,19,49–51], and the same might be expected for activation. Furthermore, different mechanisms could be required to overcome different forms of repression. Redundancy presents a substantial challenge, in part because it makes identification of factors and regulatory elements difficult. Each different type of activation may need to be characterized in isolation, before an understanding of the overall process can be obtained.

Another major challenge in understanding translational activation concerns the question of whether an activation mechanism serves as a prerequisite for translation, or is used in a spatially restricted manner. Addressing that question with *in vitro* systems would be extremely difficult, as spatial differences within the oocyte are lost in the process of preparing an extract. Our strategy of monitoring the pattern of translation *in vivo* provides a solution, and may be useful for analysis of other forms of activation.

## Materials and Methods

### Flies and transgenes

*w* ^*1118*^ flies were used as the wild type. Transgenic fly stocks were established by standard methods. Expression of the UAS transgenes was driven by the *nosGAL4VP16* [52] or *matα4-GAL-VP16* driver [53], as indicated.

The *P[UAS-GFP-MS2_18_]* and *P[UAS-GFP-boxB_6_]* transgenes are similar to *UASp-GFP-312* [54], but with 18 copies of the *MS2* binding sites or 6 copies of the *boxB* binding site instead of *mi-312* targets. *P[UAS-MCP::HA_3_::bru2^–^3^-^]* shares the same synthetic 5′ UTR as *UASp-GFP-312*, fused to MCP, 3 copies of the HA epitope, and *bru* cDNA bearing point mutations in RRM2 (K239A, F241A) and RRM3 (N521A, F523A) [27]. Mutations in the *bru* sequences were introduced using restriction sites or PCR. *P[oskT140*::*HA]* and *P[oskT140*::*GFP]* are based on a genomic DNA fragment which provides complete *osk* function and regulation [10], and have either 3 copies of the HA epitope tag or mGFP6 [55] inserted after T140. To make *P[UAS-osk1–534*:*GFP]* (which encodes a fusion protein with the first 173 amino acids of Long Osk), *P[UAS-GFP]* [26] was modified by addition of *osk* sequences that include 20 nt of the 5′ flanking region and the first 534 nt of the mRNA (the 5′ UTR and the coding region for amino acids 1–173). Derivatives with the *osk* 3′ UTR AB region [11], a version of the AB region with Bru binding sites mutated (all-) [26], and with the entire *osk* 3′ UTR were made as described [26].

For homologous recombination of *aret*, a 2.1kb region (12,270,445–12,272,531; R5.48) including the first two protein-coding exons that encode the amino terminus of female Bru (up to aa193) was targeted. Details are provided in the Supplemental Materials.

### Cloning, expression and purification of recombinant Bru and Cup proteins

GST::Bru was constructed by subcloning full-length *bru* cDNA into pGEX-2TK (GE Healthcare). GST::Cup577–947 was a gift from Robin Wharton [56]. Bru proteins for binding assays were tagged at the amino terminus with six histidine residues provided by the pET-15b (Novagen) vector and used for purification, and made use of the same mutations as in the *UAS-MCP*::*HA*::*bru* transgenes. GST fusion proteins were expressed in CodonPlus (Stratagene) *E*. *coli*, after induction with IPTG. Pelleted cells were resuspended in ice-cold GST lysis buffer (50mM Tris-Cl pH8.0, 150mM NaCl, 1mM EDTA, 1mM DTT, 2mg/ml lysozyme, and 0.1% IGEPAL-CA-630) supplemented with protease inhibitors (Complete, Mini, EDTA-free protease inhibitor cocktail tablet (Roche)), and extracts were prepared as previously described [27]. For His_6_ tagged proteins, extracts were prepared with His lysis buffer (50mM NaH_2_PO_4_H_2_O pH8.0, 300mM NaCl, 20mM imidazole, 0.01% β-Mercaptoethanol, and 2mg/ml lysozyme) supplemented with protease inhibitors. 250μl Ni-NTA Agarose (Quiagen) in 50% slurry was added per 1ml lysate, and the reaction was incubated for 1–2 hr at 4°C on a rotator. The lysate-Ni-NTA mixture was then loaded into a disposable column equilibrated with the His lysis buffer to remove flow-through and washed with increasing concentrations of imidazole in His lysis buffer (up to 40mM). The proteins were eluted with 250mM imidazole in His lysis buffer. Glycerol was added to the supernatant to 20% final volume and extracts were stored at -70°C.

### GST pull-down assay

Equivalent amounts of GST::Bru, GST::Cup or GST was first immobilized on Glutathione Sepharose 4B (GE Healthcare) prepared in 50% slurry in binding buffer (50mM Tris-Cl pH7.5, 150mM NaCl, 10% glycerol, 1mM DTT, and 0.1% IGEPAL-CA-630) supplemented with protease inhibitors, by incubating with extract overnight at 4°C on a rotator. The beads were spun down, washed and resuspended in binding buffer to make 50% slurry. Then 20μl of this slurry was incubated with ~100ng of each of the N-terminally His_6_-tagged Bru proteins in 80μl reaction containing binding buffer for 2–3 hr at room temperature with rotation. The beads were spun down, washed with binding buffer, and boiled in 5μl 2X SDS loading buffer to elute the bound proteins. Eluates were separated by SDS-PAGE and analyzed by Western blotting. The mouse anti-His antibody (ABGENT) diluted at 1:2000 and alkaline phosphatase-conjugated goat anti-mouse antibody (Applied Biosystems) diluted at 1:5000 were used to detect Bru proteins.

### *In vitro* phosphorylation assay

Phosphorylation reactions (20μl) contained ~250ng of purified substrate, 1–2 unit of recombinant PKA catalytic subunit, CK1 or CaMKII (all from NEB), and 0.2mM [γ-^32^P]ATP (adjusted to 250μCi/μmol, Perkin Elmer) in kinase buffer (buffer for PKA: 50mM Tris-Cl pH7.5, 100mM KCl, 5mM MgCl_2_, and 2.4mM DTT; buffers for CK1 or CaMKII provided by NEB) supplemented with protease inhibitors. Reactions were incubated at 30°C for 30 min and terminated by addition of 3X SDS loading buffer. Proteins were separated by SDS-PAGE, and gels were dried (Bio-Rad) and exposed to a Phosphor Screen (Molecular Dynamics) for 12 hr. The screen was then analyzed with a Typhoon laser scanner (GE Healthcare).

### RNase protection assay

RNase protection assay was performed as previously described [26], except that quantitation was done using ImageJ.

### Detection of phosphorylated Bru

Ovaries from young females, fed on yeast for 3–4 days, were dissected and extract was prepared as previously described [11] in ice-cold lysis buffer (50mM Hepes pH7.9, 150mM KCl, and 1% IGEPAL-CA-630) supplemented with protease inhibitors. Reactions (20μl) contained 10–15μg of ovary extract in phosphatase buffer (50mM Hepes pH7.9, 100mM NaCl, 10mM MgCl_2_, and 1mM DTT), and where indicated, one or more of the following components were added: 1–2 units of alkaline phosphatase (calf intestinal, NEB), 1.6M beta-glycero phosphate, and 40mM sodium vanadate. Reactions were incubated at 30°C for 1 hr and terminated by addition of 3X SDS loading buffer. Proteins were separated by SDS-PAGE and analyzed by Western blotting.

For phosphate-affinity SDS-PAGE using acrylamide-pendant Phos-tag (WAKO), 50μM Phos-tag and 200μM MnCl_2_ were added to both stacking and separating gels in solution.

### Ovary imaging

Ovaries were processed for imaging as described [26], with an alternate protocol for detection of Bru [57]. Primary antibodies were used at the following dilutions: mouse anti-HA (Covance), 1:1000; rat anti-Bru, 1:500. Secondary antibodies, used at 1:800, were Alexa Fluor 488 goat anti-mouse (Invitrogen) and Cy5 goat anti-rat. DNA was stained with TO-PRO-3 Iodide (642/661, Invitrogen) diluted 1:1000. Samples were mounted in Vectashield (Vector Labs) and analyzed with a Leica TCS-SP laser scanning confocal microscope. Quantitation of GFP levels was done using the Macnification software from images obtained using a single plane of focus. The average green fluorescence was sampled from four different regions in the nurse cell cytoplasm of each of 10 to 11, stage 5 or 6 egg chambers.

### Western blotting

Antibodies for western blotting were mouse anti-Bru (AN, unpublished) (1:8000), mouse anti-HA antibody (Covance) (1:1000), mouse anti-αTubulin (Sigma) (1:1000) and rat anti-Cup219 [58](1:2000). Alkaline phosphatase-conjugated secondary antibodies were used at 1:5000 (anti-mouse; Applied Biosystems) or 1:10000 (anti-rat; Sigma).

### RNA binding assay

The *osk* 3’ UTR *AB* probe was transcribed using MAXIscript Kit (Ambion) and uniformly labeled with [α-^32^P]UTP (800Ci/mmol, Perkin Elmer). UV cross-linking assay was performed as described [11], except that 10X binding buffer consisted of 60mM Hepes pH7.9, 300mM KCl and 20mM MgCl_2_, and was supplemented with protease inhibitors (Roche). ~500ng of purified recombinant Bru proteins was used. After electrophoresis of cross-linked adducts, gels were dried (Bio-Rad) and exposed to a Phosphor Screen (Molecular Dynamics) for 12 hr. The screen was then analyzed with a Typhoon laser scanner (GE Healthcare).

## Supporting Information

S1 Text Phosphopeptide analysis and genomic engineering of the *arrest* locus (DOCX)

S1 Table Prediction of phosphorylation sites in Bru protein (DOCX)

S1 Fig Tethered Bru proteins are stable. (A and B) Western blot of ovary extract from flies expressing MCP::HA_3_::Bru proteins as labeled. Expression of the UAS transgenes was driven by the *matα4-GAL-VP16* driver. Blots were probed with anti-HA antibody to detect Bru proteins (top) or anti-α-Tubulin antibody for loading control (bottom). All Bru fusion proteins are stable. Although the level of Δ1–146 is less than Bru+, this lower level is still sufficient for full repression by Δ334–416 and so cannot explain why Δ1–146 is impaired (Fig. 2).
(TIF)

S2 Fig Bru is phosphorylated at multiple sites and Bru phosphosilent mutations disrupt phosphorylation by PKA. (A) Phosphate-affinity SDS-PAGE using acrylamide-pendant Phos-tag that separates different phosphoprotein isoforms, followed by Western blot to detect proteins using anti-Bru antibody. When the *wild-type* ovary extract is treated with phosphatase alone, a major Bru band and two upper bands, of which one more distinct and running higher than the other, are seen. When inhibitors are also present, there is a visible smudge consisting of multiple bands above the major Bru band. Inhibitors used were sodium vanadate and beta-glycero phosphate, which are competitive inhibitors of the alkaline phosphatase. (B) *In vitro* phosphorylation assay using gamma _32_P-ATP, purified mouse PKA catalytic subunit and purified phosphosilent (Ala) mutant Bru proteins as labeled. The positions of amino acids predicted to be candidates for phosphorylation by PKA are shown in the schematic Fig. 3D. The Bru proteins are full length and used at concentrations less than the Δ334–416 Bru proteins in Fig. 3C. Top: autoradiogram to detect phosphorylation and show that compared to Bru+, S7A mutant has reduced phosphorylation. Phosphorylation of both S4A/S7A and S4A/S7A/T135A mutants is undetectable as with Δ1–146. Bottom: Western blot of proteins used in the phosphorylation assay to show the relative amounts of input proteins.
(TIF)

S3 Fig Bru is phosphorylated by PKA but not by CK1 or CaMKII. (A) *In vitro* phosphorylation assay using gamma _32_P-ATP, purified mouse PKA catalytic subunit, and purified Bru or BSA. The amount of substrates used was equivalent, but a lot higher than that shown in Fig. 3B. The autoradiogram shows that compared to BSA, Bru+ is strongly phosphorylated. (B) *In vitro* phosphorylation assay using gamma _32_P-ATP, purified rat CK1 (top) or purified rat CaMKII (middle), and purified Bru proteins as labeled. Top and middle: autoradiograms to detect phosphorylation. Both Δ334–416 and Δ1–146 Δ334–416 proteins show a low amount of phosphorylation by CK1. Bottom: Western blot of proteins used in the phosphorylation assay to show the relative amounts of input proteins. The amount of Bru+ used was equivalent in both panels.
(TIF)

S4 Fig Interaction-defective Bru mutants can repress translation of the *GFP-boxB* reporter in a tethering assay. (A-C, A’-C’) show egg chambers expressing the *GFP-boxB* reporter mRNA. (B-C, B’-C’) also express λN::HA_3_::Bru proteins, of the type shown at left. All Bru proteins include point mutations in RRM2 and RRM3 (see Fig. 2 legend). All samples were fixed in parallel and imaged together under the same settings. Expression of the UAS transgenes was driven by the *matα4-GAL-VP16* driver. (D) GFP fluorescence was quantitated using Macnification. The mean was calculated from over 20 samples per genotype. (E) RNase protection assays: *GFP-boxB* RNA levels were quantified by ImageJ and normalized using the *rp49* signal. The value for none, which lacks any λN::HA_3_::Bru proteins, was set to one. The mean and standard deviation were calculated from three independent experiments.
(TIF)

S5 Fig RNA-binding activity of mutant Bru proteins. (A) UV-crosslinking assay of Bru binding to the radiolabeled *osk* 3′ UTR AB region RNA. The Bru proteins used are indicated above the autoradiogram showing cross-linked Bru. At bottom is a western blot of input proteins showing the relative amounts used in the assay. (B) RNA-binding activity and Bru protein levels were quantitated using ImageJ. The RNA binding was normalized for the protein level, and the value for Bru+ was set to one. The mean and SEM were calculated from three independent experiments. The change in RNA-binding activity in a pair-wise comparison was considered significant in S4/S7 and S4/S7/T135 using the student’s T test (*p≤0.05; **p≤0.01). The change in RNA binding of S4E/S7E (26% decrease from the ala counterpart with p = 0.008) was considered more statistically significant than that of S4E/S7E/T135E (40% decrease from the ala counterpart with p = 0.04), due to a greater sample variation of S4/S7/T135.
(TIF)

S6 Fig Bru expression pattern and level in *aret* mutants. (A-F) show egg chambers stained for Bru and derived from flies with a distinct, genetically engineered *aret* gene, as labeled. All have a single copy of *aret* in *trans* to *Df(2L)aret*. (A-C) are stage 7, and (D-F) are stage 9 egg chambers. All samples were fixed in parallel and imaged together under the same settings. (G) Western blot of ovary extract from flies with a distinct, genetically engineered *aret* gene, as labeled. Blots were probed with anti-Bru antibody to detect Bru proteins (top) or anti-α-Tubulin antibody for loading control (bottom).
(TIF)
