# Supplementary material for: Region-Specific Activation of oskar mRNA Translation by Inhibition of Bruno-Mediated Repression
Source: PLoS Genet. 2015 Feb 27;11(2):e1004992. doi: 10.1371/journal.pgen.1004992 (PMC4344327; doi:10.1371/journal.pgen.1004992)
Supplement: S1 Text — (DOCX) [file pgen.1004992.s001.docx]

Phosphopeptide Analysis of His_6_-Tagged Bruno 1-148

Experimental: Trypsin in-gel digest was performed and followed by desalting with a µC18 ziptip (Millipore). The samples were run by LC-MS/MS on a Thermo Orbitrap Elite mass spectrometer equipped with an ultra-high-pressure Dionex Ultimate 3000 RSLC nano-LC system with buffer A (0.1% (v/v) formic acid in water) and buffer B (0.1% (v/v) formic acid in acetonitrile). Peptides were concentrated onto an in-house packed 100-nm-inner diameter x 2-cm C18 column (Magic C18, 3 µm, 100 Å, Michrom Bioresources Inc.), and then separated on a 75-nm-inner diameter x 15-cm C18 fused silica column (Dionex). Liquid chromatography was performed using a gradient of 3 to 27 to 45% buffer B over 0 to 69 to 80 min, with a total run time of 98 min. Data-dependent acquisition in the orbitrap detector used scan cycles of one MS1 scan (300-1700) at a resolution of 60,000 followed by MS2 at resolution 15,000 on the 10 most intense ions using CID fragmentation with 3 *m/z* isolation width and normalized collision energy of 35. Singly charged ions were excluded. Dynamic exclusion was used with 1 repeat count, 24 sec repeat duration, 12 sec exclusion duration and exclusion list of 500 ions. Technical replicates were performed for each sample.

Peptide/protein identification was performed with ProteomeDiscoverer 1.4 using search nodes SEQUEST_HT (Thermo) and MS-Amanda (Protein Chemistry Facility, Vienna, Austria, http://ms.imp.ac.at/?goto=pd-nodes). The initial search was conducted with SwissProt database (Jan 2014 version with 456701 sequences) and the search parameters used were as follows: two missed cleavages were permitted, static modifications on cysteine carbamidomethylation, dynamic modifications on oxidized methionine, 10 ppm precursor tolerance and 0.02 Da MS/MS tolerance. Peptide identifications were filtered using the Target Decoy validation node where decoy database of reversed sequences is generated for calculating false discovery rates. A high confidence FDR 1% filtering was applied. The contaminant proteins were determined from the initial search, and then a custom database of 10-contaminant trypsin and human keratin protein sequences, and the Bruno sequence were concatenated with the E. coli database (from Jan 14, 2014) to create a custom database with 4963 sequences. The data was searched with the custom and a matched decoy database using the same parameters as before with the addition of dynamic modification of phosphorylation of serine and threonine and maximum 2 modifications/peptide. The search results were validated with the target decoy and, in parallel, routed to phosphoRS v. 3.0, where scores above 50 are considered good scores. PhosphoRS also assigns a probability for likelihood of a particular phosphorylation site on a given peptide on those peptide identifications made using SEQUEST HT. The results of all searches were filtered for high confidence peptides (FDR 1%), with 2 peptides/protein.

Results: Bruno was observed with 3657 spectral counts from the LC-MS/MS runs searched with SEQUEST_HT and MS-Amanda, along with 27 contaminant proteins having much lower spectral counts. Overexpressed Bruno with His_6_ tag had spectral counts covering 50% of the sequence. There is one sequence stretch not amenable to proteolytic digest, which was not observed, but all other peptides were observed with tens of spectral counts. Several phosphopeptides were observed with high confidence only in the kinase-treated sample. (6)ASFLANRR(13) was observed as a phosphopeptide in 3 spectral counts, and the site assignment of phosphorylation of S7 is unambiguous due to the single serine residue. The (6)ASFLANR(12) S7 phosphopeptide is also detected. The peptides (-3)GSHMFTSRASFLANR(12) and (-3)GSHMFTSRASFLANR(13) are also detected as phosphopeptides at high confidence. In this case the site assignment is ambiguous, with phosphoRS assigning equal probability for phosphorylation on T3, S4, and S7. Further examination of the SEQUEST_HT results reveals that 8 of the 17 MS/MS have a higher XCorr value for the S7 site assignment than any other assignment, and 7 have equivalent XCorr (scores differ by < 0.1) for the T3, S4 and S7 or the S4 and S7 assignments. Thus, the parsimonious explanation of the data restricts the assignment of phosphorylation to S7, though it does not rule out the possibility of phosphorylation of S4 and T3. In addition, at least 2 phosphorylation sites are detected on the 18-amino-acid, His-tag sequence in one or both samples.

Genomic Engineering of the *arrest* Locus

Since the entire *aret* locus is too big to target (over 100kb), we targeted a 2.1kb region (12,270,445 to12,272,531; the gene annotation based on Release 5.48) including the first two protein-coding exons that encode the amino terminus of female Bru (up to aa193). First the founder knock-out line was made by a modified ends-out targeting. The targeting donor DNA fragment contained 5’ and 3’ homologous arms flanking the targeted region, and a loxP-flanked *white+* (*w+*) transgenic marker juxtaposed by an attP site of *Φ*C31, as in pGX-attP (Huang et al., 2009). The 5’ arm (12,265,973 to 12,270,438) was made by PCR using the *w1118* genomic DNA. The 4.5kb 5’ arm was cloned in three fragments into pCRII-TOPO vector (Invitrogen). The 5’-most fragment was amplified to introduce a 5’ NotI site and included the KpnI site near the 3’ end. The middle fragment was amplified to include the KpnI near the 5’ end and the AflII near the 3’ end. The 3’-most fragment was amplified to introduce a 3’ KpnI site and included the AflII site near the 5’ end. Finally, these three fragments were ligated together into pGX-attP using NotI and KpnI within the 5’ MCS.

The 3’ arm (12,272,538 to 12,276,637) was made by PCR using p4045 as a template. The plasmid was modified by converting XhoI at the 5’ end of the 3’ arm sequence into XbaI by cutting with XhoI, filling in the ends, and ligating with XbaI linker. Similarly, the plasmid was further modified by converting AflIII at the 3’ end of the 3’ arm sequence into BamHI. Then the XbaI-BamHI fragment was cloned into pGX-attP using SpeI and BglII within the 3’ MCS.

Transgenic stocks were established by standard methods. Virgins carrying the targeting donor DNA were crossed to males carrying *hs-flp* and *hs-ISceI* (Bloomington 6935), and the resulting progeny were heat-shocked three times over three days as embryos. Many mosaic-eyed progeny were obtained, indicating ends-out targeting worked. Then the mosaic-eyed virgins were again crossed to males carrying *P{70flp}10* (Bloomington 6938) in order to reduce the number of false positives. The resulting progeny were heat-shocked once three days later as embryos. Red-eyed males were selected and balanced over CyO chromosome after segregation testing. Homozygous females were sterile, as expected for *aret* phenotype. Deletion was further verified by PCR.

The *w+* marker in between the 5’ and 3’ arms was removed by crossing the knock-out males to virgins carrying *hs-Cre* (Bloomington 1092). There was no heat shock involved because *Cre* was supposed to be constitutively active. The desired progeny, which have the knock-out chromosome and *hs-Cre*-bearing chromosome, were orange-eyed due to the marker present in *hs-Cre*. The orange-eyed males were then crossed to virgins carrying *vasa-ΦC31* (Bloomington 24749). Balanced stocks were established from a single male and sent for injection with various replacement plasmids in pGE-attB vector. The *w+* marker and extra vector sequences were removed by Cre recombinase, as described above, and balanced stocks were established from a single male. Correct integration and presence of appropriate mutations were verified by PCR.

To produce *aret* mutants containing triple *ala* or *glu* mutations, we performed site-directed mutagenesis with two rounds of PCR for each of *S4/S7* and *T135* mutations. Each of the two PCR fragments were synthesized in the first PCR reaction using a mutagenic primer, a primer that anneals to either the 5’ or 3’ end of the replacement DNA, and *w1118* DNA as the template. Terminal restriction sites, EcoRI (5’) and KpnI (3’), were introduced. The two PCR fragments were purified, and a second PCR was performed using a mix of these two fragments and the two terminal primers used previously. The final mutagenized PCR fragment (~2.1kb) was first cloned into pCRII-TOPO, and EcoRI-KpnI fragment was subcloned into pSP73. Internal EcoRV site (12,271,334) was used to cut out and ligate the 5’ EcoRI-EcoRV fragment carrying *S4/S7* mutation, and the 3’ EcoRV-KpnI fragment carrying *T135* mutation, into pGE-attB vector using EcoRI and KpnI.

All PCR reactions were carried out using Phusion High-Fidelity PCR Master Mix (Finnzymes) to minimize the error rate.
