# Supplementary material for: Region-Specific Activation of oskar mRNA Translation by Inhibition of Bruno-Mediated Repression
Source: PLoS Genet. 2015 Feb 27;11(2):e1004992. doi: 10.1371/journal.pgen.1004992 (PMC4344327; doi:10.1371/journal.pgen.1004992)
Supplement: S1 Table — (DOCX) [file pgen.1004992.s002.docx]

KinasePhos (<http://kinasephos.mbc.nctu.edu.tw/>) predictions.

The prediction specificity value was set to 90%.

Location (AA) Bit Score E-value Kinases

7 -1.8 22 PKC

7 4.5 1.4 PKA

7 -3.4 52 CaM-II

7 -8.7 97 PKG

7 -3.9 89 IKK

7 -5.9 96 PKB

26 0.1 8.4 EGFR

26 -3.0 33 Syk

26 -2.8 29 Jak

33 -7.0 94 ATM

36 -3.3 35 CKII

36 -1.1 21 IKK

36 3.3 1.7 Other_MDD

37 -3.8 46 PKC

37 -4.6 57 CKII

37 -9.7 1e+02 PKG

37 -3.3 74 CKI

37 -7.0 1e+02 cdc2

37 -2.5 50 IKK

38 0.1 10 PKC

38 -0.1 7.2 CKII

38 -7.0 1e+02 CaM-II

38 -3.0 66 CKI

38 -7.1 1e+02 cdc2

38 -3.0 46 PKB

42 -7.9 92 PKG

42 -4.9 98 CKI

42 -0.2 11 IKK

42 0.8 4 Other_MDD

46 -1.1 23 cdc2

46 -4.4 55 MAPK

46 3.6 2.6 CDK

46 -6.8 93 ATM

46 3.5 1.5 Other_MDD

46 -0.8 14 Other_MDD

46 -8.2 70 MAPK

69 -9.1 1e+02 cdc2

73 -3.3 73 CKI

73 -5.4 91 PKB

84 -3.6 82 CKI

84 -9.9 1e+02 cdc2

88 -9.8 1e+02 cdc2

135 -2.8 20 PKA

138 -1.8 32 Other_MDD

140 -4.3 73 PKC

140 -6.4 48 MAPK

161 -6.3 86 MAPK

161 -1.8 34 IKK

165 -8.2 94 PKG

165 -1.7 19 ATM

188 -4.2 36 PKA

201 -1.3 22 Other_MDD

232 -3.6 84 CKI

287 -1.5 25 Other_MDD

293 -6.3 70 PKG

300 -0.3 9.9 ATM

310 -1.6 28 cdc2

310 -0.4 11 MAPK

310 1.1 3.3 CDK

310 0.7 9.2 Other_MDD

319 -6.9 1e+02 PKC

319 -3.2 35 CKII

319 0.0 8.4 Other_MDD

349 -9.0 79 MAPK

351 -1.8 33 Other_MDD

352 -2.0 27 PKC

352 -4.7 97 CKI

352 -7.9 1e+02 cdc2

352 -4.2 94 IKK

354 -6.9 1e+02 PKC

355 -2.5 22 CDK

366 -6.2 96 cdc2

366 -4.6 57 MAPK

366 -1.2 7.5 CDK

366 -3.4 76 IKK

366 -5.4 90 PKB

375 -6.8 53 MAPK

406 -1.4 26 IKK

407 -4.5 55 CKII

407 -3.3 74 IKK

408 -2.7 37 PKC

408 0.1 6.5 CKII

408 -4.5 97 CKI

408 -7.3 1e+02 cdc2

408 -6.3 86 ATM

408 -2.9 63 IKK

408 -0.1 11 PKB

408 -0.6 13 Other_MDD

422 -1.8 13 MAPK

422 -0.8 11 Other_MDD

436 -5.5 94 PKC

436 -5.5 57 CDK

439 -0.9 16 Other_MDD

441 -5.8 79 ATM

451 -5.6 95 PKC

451 -9.5 85 MAPK

452 -4.5 74 cdc2

452 -0.5 12 MAPK

452 -2.7 17 CDK

452 -2.4 12 CDK

452 -6.6 91 ATM

452 1.3 7.5 Other_MDD

452 -7.5 61 MAPK

455 -9.8 1e+02 cdc2

455 -1.9 16 CDK

455 -4.9 98 IKK

459 -9.4 84 MAPK

474 0.0 6.1 PKA

476 -4.5 97 INSR

478 -6.8 1e+02 INSR

488 -9.4 1e+02 cdc2

488 -3.5 80 IKK

490 -9.5 1e+02 cdc2

490 -3.7 85 IKK

493 -8.2 94 PKG

493 -5.2 98 CKI

493 -6.5 88 MAPK

494 -9.8 1e+02 PKG

494 -7.4 85 CDK

494 -1.8 31 Other_MDD

495 -5.4 92 CaM-II

495 -9.8 1e+02 PKG

495 -1.3 25 cdc2

495 1.5 4.7 MAPK

495 -1.2 11 CDK

495 -2.3 11 CDK

495 -4.1 49 ATM

495 -0.9 18 IKK

495 2.7 5.1 Other_MDD

499 -5.4 92 CaM-II

499 -4.9 63 MAPK

503 -4.3 52 CKII

503 -3.3 27 PKG

503 -9.6 1e+02 cdc2

525 -7.2 1e+02 INSR

532 -2.8 28 CKII

548 -9.6 1e+02 PKG

558 -3.4 34 PKC

573 1.0 5.1 Other_MDD

588 -6.5 1e+02 PKC

NetPhosK (<http://www.cbs.dtu.dk/services/NetPhosK/>) predictions.

The method used was “prediction without Evolutionary Stable Sites filtering” with a threshold value of 0.5.

Location (AA) Score Kinase

3 0.83 PKC

4 0.5 PKA

4 0.52 cdc2

7 0.64 PKC

19 0.63 PKC

26 0.51 SRC

26 0.55 EGFR

26 0.52 INSR

33 0.6 PKC

37 0.65 PKC

42 0.58 DNAPK

42 0.55 PKC

46 0.55 GSK3

46 0.64 cdk5

68 0.6 PKC

73 0.59 DNAPK

84 0.53 CKI

88 0.56 PKA

88 0.55 cdc2

95 0.84 PKC

98 0.5 CKI

98 0.5 cdc2

135 0.6 PKA

140 0.62 CKII

140 0.7 PKC

161 0.6 CKII

165 0.62 DNAPK

165 0.64 ATM

174 0.53 SRC

188 0.87 PKC

191 0.61 PKA

191 0.51 cdc2

277 0.53 cdc2

284 0.53 PKC

287 0.66 PKC

293 0.82 PKC

298 0.72 PKC

300 0.64 DNAPK

300 0.57 ATM

300 0.68 PKC

310 0.53 p38MAPK

319 0.52 CKII

319 0.72 PKC

352 0.53 cdc2

355 0.56 p38MAPK

355 0.53 cdk5

366 0.53 cdk5

399 0.59 cdc2

406 0.52 cdc2

408 0.57 cdc2

413 0.53 cdc2

422 0.6 p38MAPK

436 0.6 DNAPK

436 0.5 PKC

452 0.53 p38MAPK

452 0.52 cdk5

467 0.57 cdc2

488 0.56 cdc2

490 0.5 cdc2

494 0.8 PKC

495 0.52 cdc2

495 0.51 GSK3

495 0.55 cdk5

503 0.5 cdc2

534 0.51 CKII

539 0.61 PKC

557 0.8 PKC

560 0.54 cdc2

588 0.87 PKC

601 0.75 PKA

601 0.54 PKG
