# Supplementary figures and images for: Region-Specific Activation of oskar mRNA Translation by Inhibition of Bruno-Mediated Repression
Source: PLoS Genet. 2015 Feb 27;11(2):e1004992. doi: 10.1371/journal.pgen.1004992 (PMC4344327; doi:10.1371/journal.pgen.1004992)

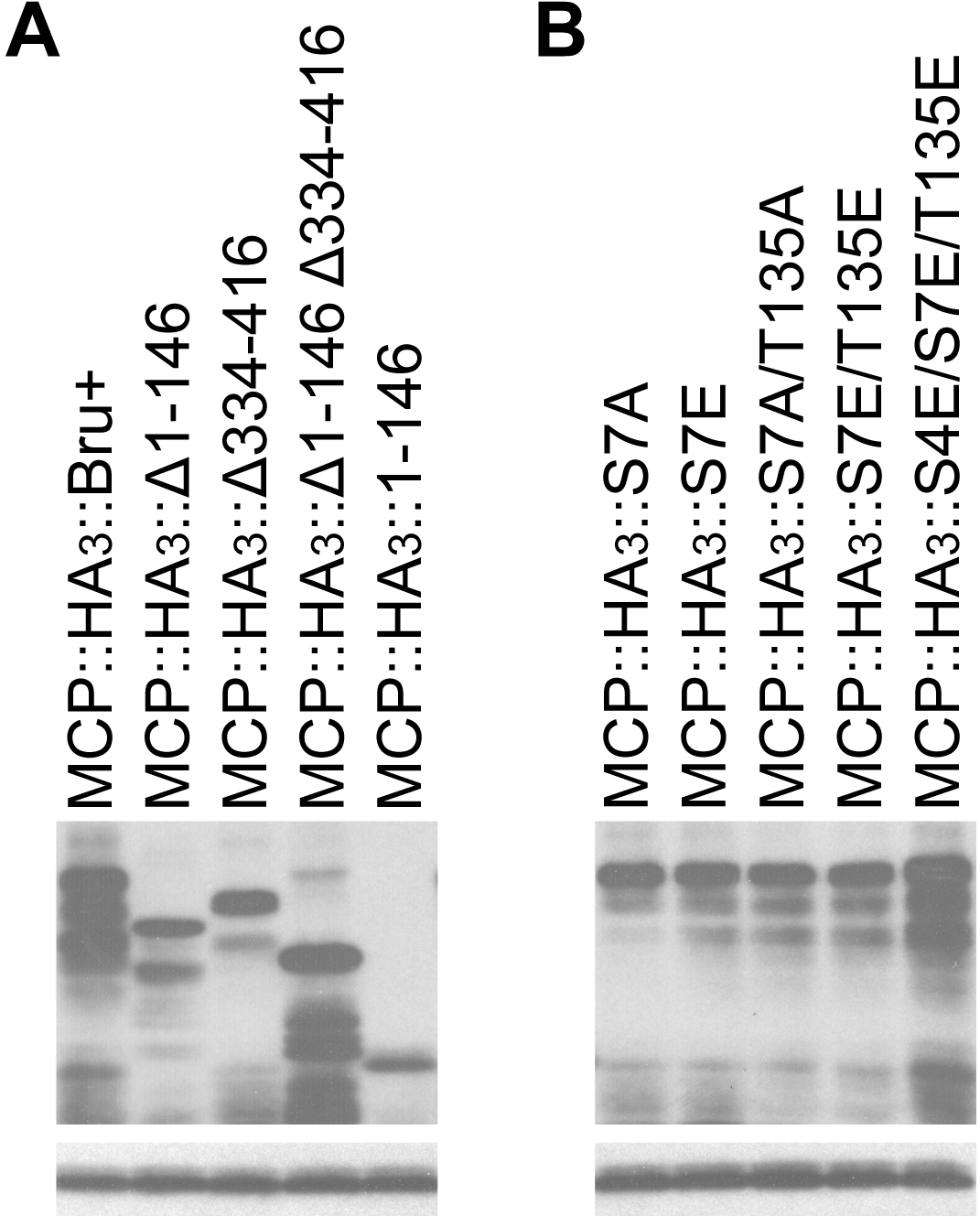

Supplement: S1 Fig — (A and B) Western blot of ovary extract from flies expressing MCP::HA3::Bru proteins as labeled. Expression of the UAS transgenes was driven by the matα4-GAL-VP16 driver. Blots were probed with anti-HA antibody to detect Bru proteins (top) or anti-α-Tubulin antibody for loading control (bottom). All Bru fusion proteins are stable. Although the level of Δ1–146 is less than Bru+, this lower level is still sufficient for full repression by Δ334–416 and so cannot explain why Δ1–146 is impaired (Fig. 2). (TIF) [file pgen.1004992.s003.tif]

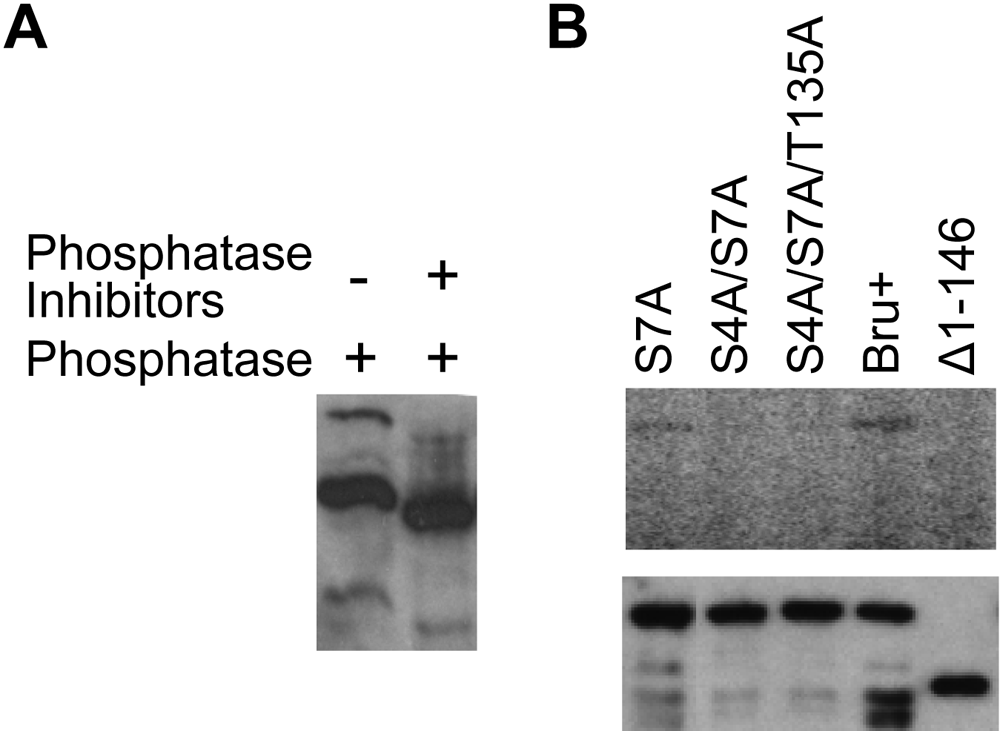

Supplement: S2 Fig — (A) Phosphate-affinity SDS-PAGE using acrylamide-pendant Phos-tag that separates different phosphoprotein isoforms, followed by Western blot to detect proteins using anti-Bru antibody. When the wild-type ovary extract is treated with phosphatase alone, a major Bru band and two upper bands, of which one more distinct and running higher than the other, are seen. When inhibitors are also present, there is a visible smudge consisting of multiple bands above the major Bru band. Inhibitors used were sodium vanadate and beta-glycero phosphate, which are competitive inhibitors of the alkaline phosphatase. (B) In vitro phosphorylation assay using gamma 32P-ATP, purified mouse PKA catalytic subunit and purified phosphosilent (Ala) mutant Bru proteins as labeled. The positions of amino acids predicted to be candidates for phosphorylation by PKA are shown in the schematic Fig. 3D. The Bru proteins are full length and used at concentrations less than the Δ334–416 Bru proteins in Fig. 3C. Top: autoradiogram to detect phosphorylation and show that compared to Bru+, S7A mutant has reduced phosphorylation. Phosphorylation of both S4A/S7A and S4A/S7A/T135A mutants is undetectable as with Δ1–146. Bottom: Western blot of proteins used in the phosphorylation assay to show the relative amounts of input proteins. (TIF) [file pgen.1004992.s004.tif]

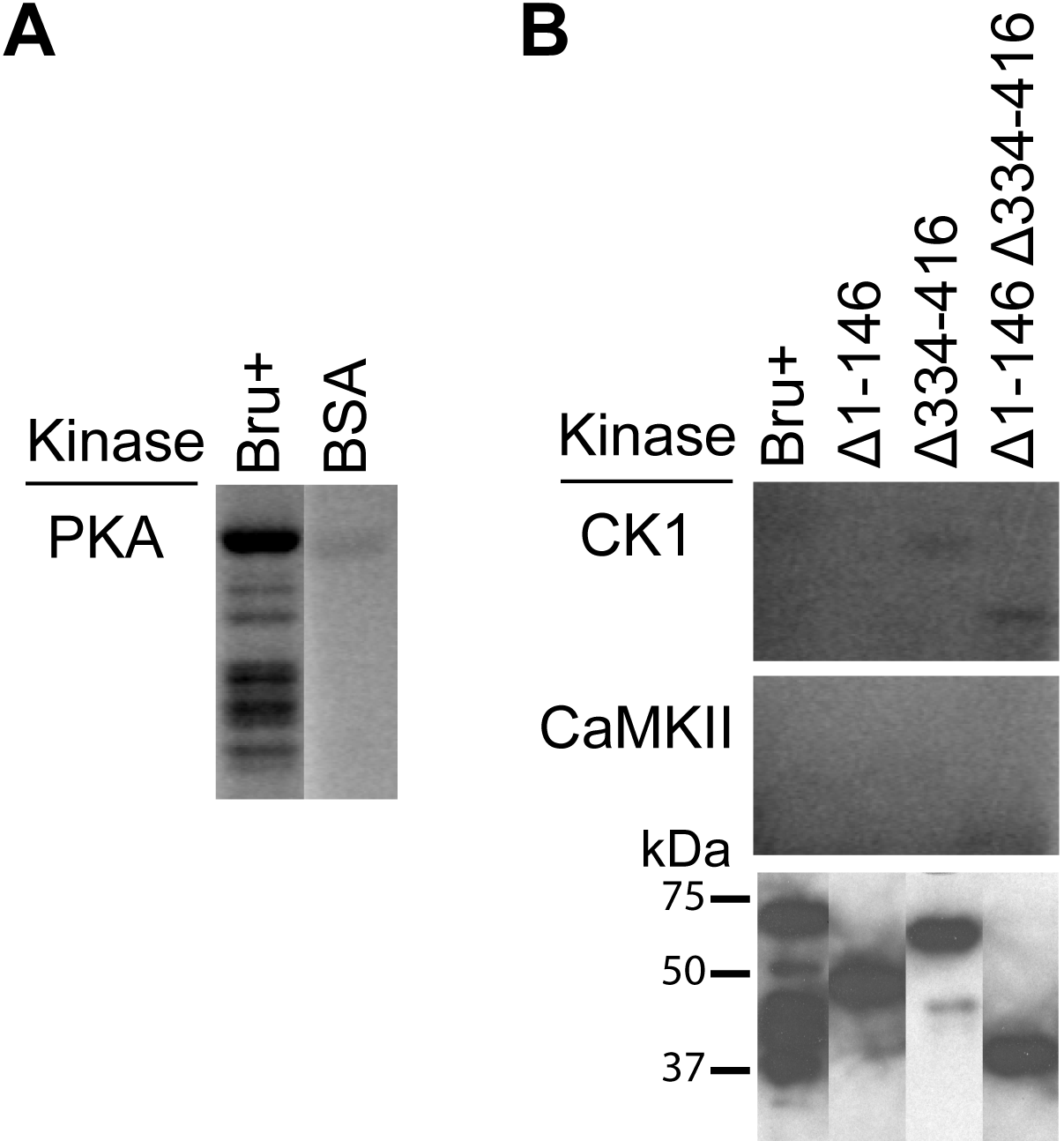

Supplement: S3 Fig — (A) In vitro phosphorylation assay using gamma 32P-ATP, purified mouse PKA catalytic subunit, and purified Bru or BSA. The amount of substrates used was equivalent, but a lot higher than that shown in Fig. 3B. The autoradiogram shows that compared to BSA, Bru+ is strongly phosphorylated. (B) In vitro phosphorylation assay using gamma 32P-ATP, purified rat CK1 (top) or purified rat CaMKII (middle), and purified Bru proteins as labeled. Top and middle: autoradiograms to detect phosphorylation. Both Δ334–416 and Δ1–146 Δ334–416 proteins show a low amount of phosphorylation by CK1. Bottom: Western blot of proteins used in the phosphorylation assay to show the relative amounts of input proteins. The amount of Bru+ used was equivalent in both panels. (TIF) [file pgen.1004992.s005.tif]

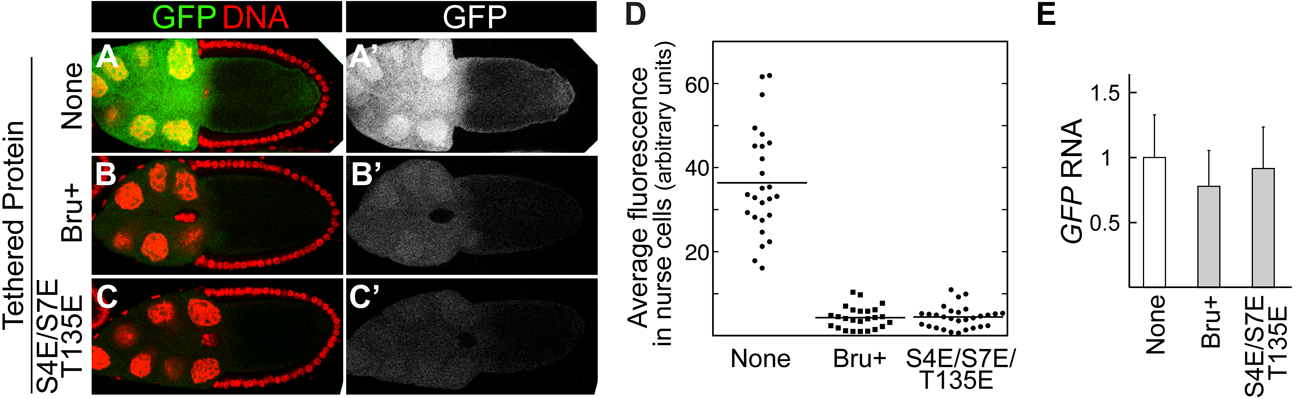

Supplement: S4 Fig — (A-C, A’-C’) show egg chambers expressing the GFP-boxB reporter mRNA. (B-C, B’-C’) also express λN::HA3::Bru proteins, of the type shown at left. All Bru proteins include point mutations in RRM2 and RRM3 (see Fig. 2 legend). All samples were fixed in parallel and imaged together under the same settings. Expression of the UAS transgenes was driven by the matα4-GAL-VP16 driver. (D) GFP fluorescence was quantitated using Macnification. The mean was calculated from over 20 samples per genotype. (E) RNase protection assays: GFP-boxB RNA levels were quantified by ImageJ and normalized using the rp49 signal. The value for none, which lacks any λN::HA3::Bru proteins, was set to one. The mean and standard deviation were calculated from three independent experiments. (TIF) [file pgen.1004992.s006.tif]

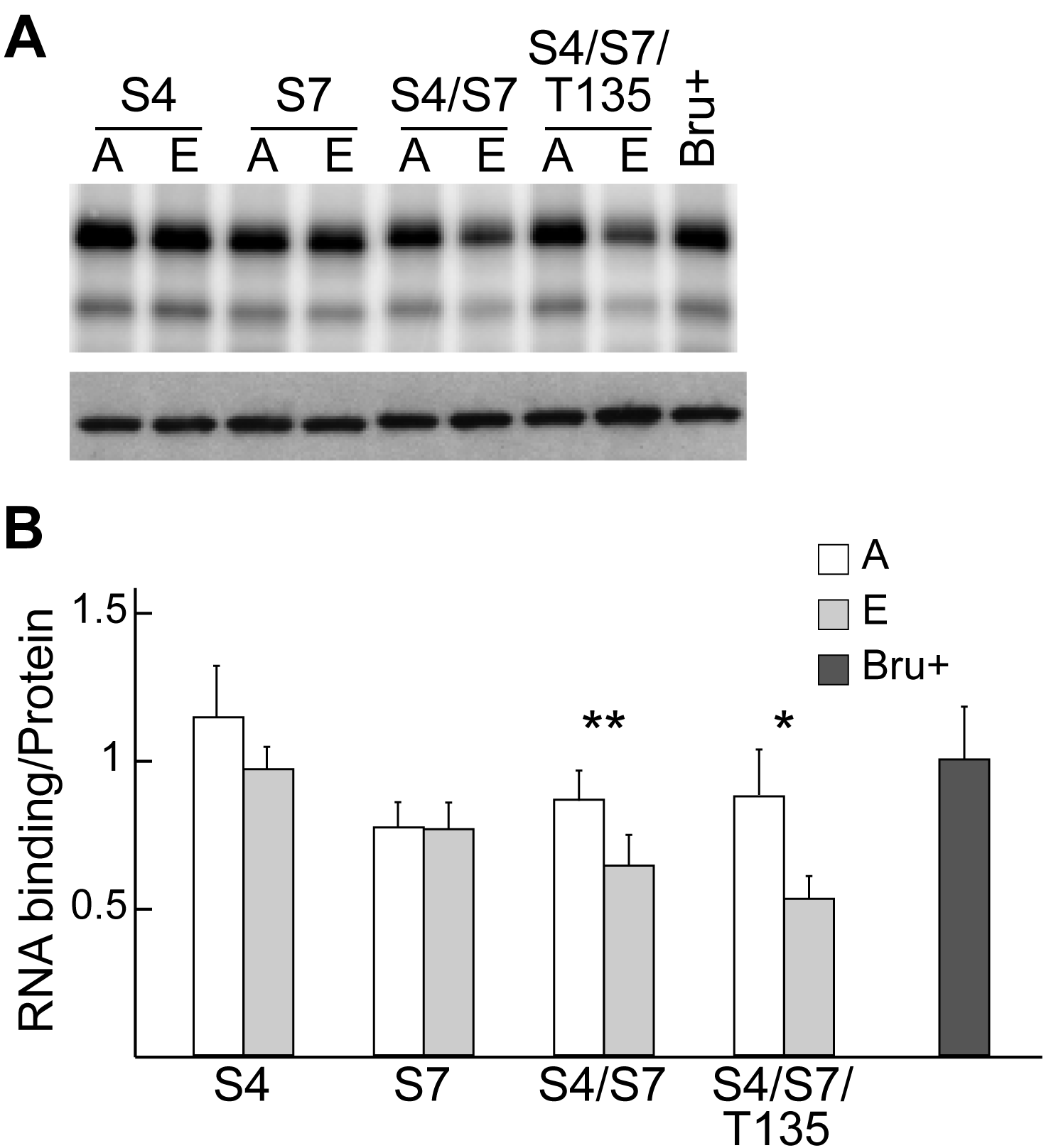

Supplement: S5 Fig — (A) UV-crosslinking assay of Bru binding to the radiolabeled osk 3′ UTR AB region RNA. The Bru proteins used are indicated above the autoradiogram showing cross-linked Bru. At bottom is a western blot of input proteins showing the relative amounts used in the assay. (B) RNA-binding activity and Bru protein levels were quantitated using ImageJ. The RNA binding was normalized for the protein level, and the value for Bru+ was set to one. The mean and SEM were calculated from three independent experiments. The change in RNA-binding activity in a pair-wise comparison was considered significant in S4/S7 and S4/S7/T135 using the student’s T test (*p≤0.05; **p≤0.01). The change in RNA binding of S4E/S7E (26% decrease from the ala counterpart with p = 0.008) was considered more statistically significant than that of S4E/S7E/T135E (40% decrease from the ala counterpart with p = 0.04), due to a greater sample variation of S4/S7/T135. (TIF) [file pgen.1004992.s007.tif]

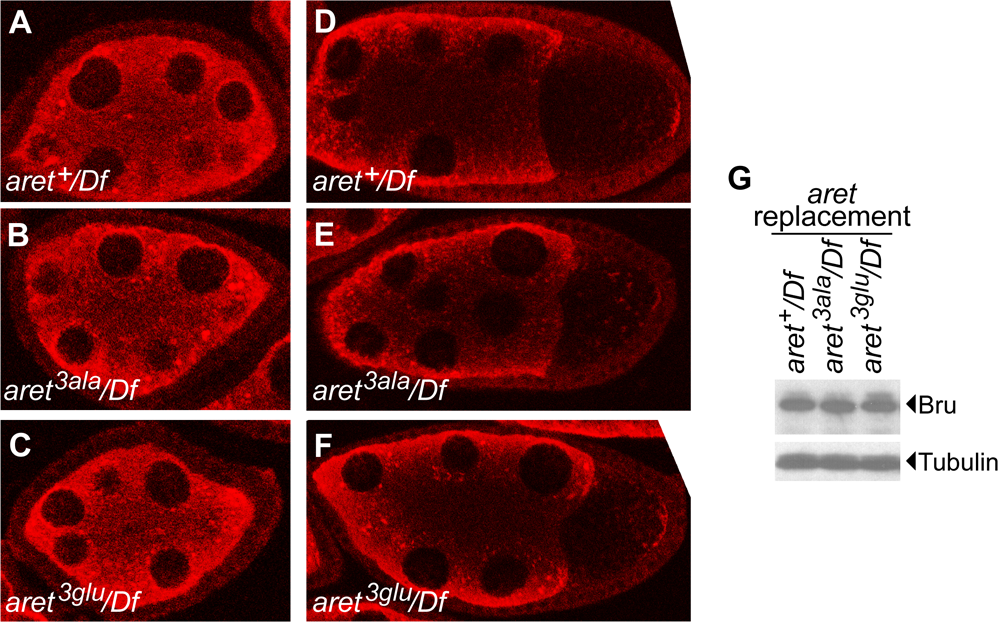

Supplement: S6 Fig — (A-F) show egg chambers stained for Bru and derived from flies with a distinct, genetically engineered aret gene, as labeled. All have a single copy of aret in trans to Df(2L)aret. (A-C) are stage 7, and (D-F) are stage 9 egg chambers. All samples were fixed in parallel and imaged together under the same settings. (G) Western blot of ovary extract from flies with a distinct, genetically engineered aret gene, as labeled. Blots were probed with anti-Bru antibody to detect Bru proteins (top) or anti-α-Tubulin antibody for loading control (bottom). (TIF) [file pgen.1004992.s008.tif]
